# Phosphatidic acid phosphatase LPIN1 in phospholipid metabolism and stemness in hematopoiesis and AML

**DOI:** 10.1002/hem3.70118

**Published:** 2025-04-22

**Authors:** Karin Huber, Swati Garg, Lena Schlautmann, Rui Wang, Lixiazi He, Richard Huth, Alireza Pouya, Christian Rohde, Maike Janssen, Christian Lüchtenborg, Christian Arnold, Pilar M. Luque‐Navarro, Judith B. Zaugg, Simon Raffel, Carsten Müller‐Tidow, Irmela Jeremias, Luisa C. López‐Cara, Britta Brügger, Caroline Pabst

**Affiliations:** ^1^ Department of Medicine V, Hematology, Oncology and Rheumatology University Hospital Heidelberg Heidelberg Germany; ^2^ Department of Medicine II, Hematology and Oncology University Hospital Schleswig Holstein Campus Kiel Germany; ^3^ Department of Medical Oncology Dana Farber Cancer Institute Boston Massachusetts USA; ^4^ Molecular Medicine Partnership Unit (MMPU) University of Heidelberg and European Molecular Biology Laboratory (EMBL) Heidelberg Germany; ^5^ Heidelberg University Biochemistry Center (BZH) Heidelberg Germany; ^6^ European Molecular Biology Laboratory (EMBL) Heidelberg Germany; ^7^ Department of Pharmaceutical and Organic Chemistry, Faculty of Pharmacy University of Granada Granada Spain; ^8^ Research Unit Apoptosis in Hematopoietic Stem Cells Helmholtz Munich Oberschleißheim Germany; ^9^ Department of Pediatrics Dr. Von Hauner Children's Hospital, LMU University Hospital LMU Munich Munich Germany; ^10^ German Cancer Consortium (DKTK), Partner Site Munich Munich Germany

## Abstract

Targeting metabolism represents a promising approach to eradicate leukemic stem cells (LSCs) that are considered critical drivers of relapse in acute myeloid leukemia (AML). In this study, we demonstrate that the phosphatidic acid phosphatase LPIN1, which regulates the synthesis of diacylglycerol, the key substrate for triacylglycerol, and phospholipid production, is crucial for the function of healthy and leukemic hematopoietic stem and progenitor cells (HSPC and LSC). *LPIN1* mRNA was highly expressed in the CD34+ compartment of primary human AML samples. *LPIN1* suppression inhibited the proliferation of primary leukemic cells and normal HSPCs in vitro and in xenotransplantation assays. Lipidomics analyses revealed a reduction of phosphatidylcholine (PC) and phosphatidylethanolamine and an upregulation of sphingomyelin upon *LPIN1* depletion. Distinct phospholipid composition was associated with genetic AML groups, and targeting PC production by choline kinase inhibitors showed strong anti‐leukemic activity. In summary, our data establish a regulatory role of *LPIN1* in HSPC and LSC function and provide novel insights into the role of glycerophospholipid homeostasis in stemness and differentiation.

## Graphical abstract

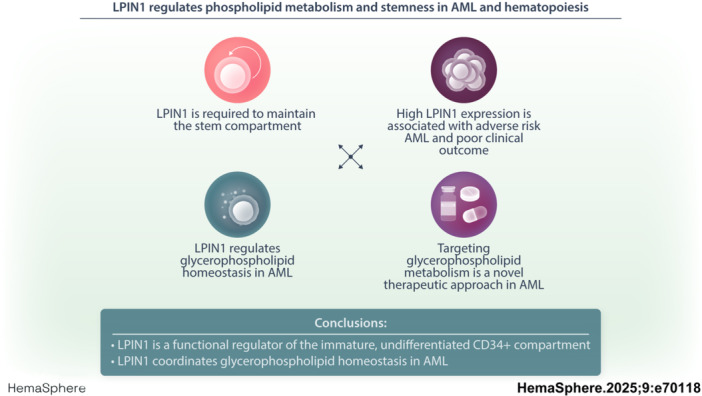

## INTRODUCTION

Prognosis in acute myeloid leukemia (AML) remains poor, especially for elderly patients.^1^ Therapy resistance is a frequent phenomenon often caused by residual leukemic stem cells (LSCs) that escape chemotherapy due to their stem cell‐like characteristics that distinguish them from bulk leukemic blasts.^2^, ^3^ Besides, LSCs were shown to exhibit distinct metabolic characteristics.^4^, ^5^, ^6^ LSCs depend on metabolizing amino acids for energy supply and low reactive oxygen species (ROS) levels.^5^, ^7^, ^8^, ^9^ Newer studies showed that certain LSCs favor fatty acids over amino acids for ATP synthesis.^5^, ^7^, ^10^ However, the role of lipids and lipid signaling in AML remains incompletely understood.

*LPIN1* was first identified in fatty liver dystrophy (fld) mice.^11^ Due to a spontaneous mutation in the *LPIN1* gene, this mouse strain suffers from metabolic abnormalities such as loss of body fat, elevated triacylglycerol (TG) levels, insulin resistance, and peripheral neuropathy.^12^, ^13^, ^14^ The translation product of *LPIN1* is the magnesium (Mg2+)‐dependent phosphatidic acid phosphatase (PAP) LPIN1.^15^ Together with LPIN2 and LPIN3, it forms a family of three PAP enzymes that catalyze the dephosphorylation of phosphatidic acid (PA), resulting in the generation of diacylglycerol (DG).^16^ By controlling DG levels, LPIN1 regulates the production of downstream metabolites including TG as energy storage, membrane lipids, and downstream signaling molecules such as mammalian target of rapamycin (mTOR), phospholipase C (PLC), or sphingosine‐kinase‐1.^17^, ^18^, ^19^ Furthermore, LPIN1 also acts as a transcriptional coactivator.^20^ By interaction with transcription factors such as peroxisome proliferator‐activated receptor alpha (PPARA) and its coactivator PPARG coactivator‐1 alpha (PGC‐1A), or with histone acetyltransferases such as p300, LPIN1 supports transcription of genes for fatty acid oxidation.^20^, ^21^ *LPIN1* overexpression has been observed in an increasing number of cancers including breast cancer, high‐grade prostate cancer, and lung adenocarcinoma. In the latter two and triple‐negative breast cancer, it is associated with an adverse prognosis, while association with a favorable course of disease is assumed for other breast cancer entities.^22^, ^23^, ^24^, ^25^ A reduction of *LPIN1* expression decreased the migration of prostate cancer cells via the activation of RhoA and also decreased the migration of breast cancer cells.^22^, ^25^ In triple‐negative breast cancer and lung adenocarcinoma reduced expression of *LPIN1* led to endoplasmic reticulum (ER) stress, thereby activating the inositol‐requiring enzyme‐1 alpha (IRE‐A) pathway.^23^, ^24^ To date, the role of *LPIN1* in hematopoietic malignancies remains elusive. In this study, we investigated the function of *LPIN1* in steady state normal hematopoiesis and AML.

## MATERIALS AND METHODS

### Patient and cord blood samples

AML patient samples and cord blood units were collected upon receipt of written informed consent in accordance with the Declaration of Helsinki. The project was approved by the Research Ethics Board of the Medical Faculty of Heidelberg University. Cord blood units were collected at the Department of Obstetrics at University Hospital Heidelberg. Patient‐derived xenograft AML‐491, its relapse sample AML‐661, AML‐602, AML‐663, AML‐346, and AML‐372 cells were a kind gift of I. Jeremias and B. Vick and generated as described via serial transplantation of primary patient leukemia cells into NOD.Cg‐*Prkdc* ^*scid*^ *II2rg* ^*tm1Wjl*^ (NSG) mice.^26^

### Xenotransplantation

NOD.Cg‐*Prkdc* ^*scid*^ *Il2rg* ^*tm1Wjl*^ (NSG) mice were obtained from Jackson Laboratories. NOD.Cg‐*Kit* ^*W‐41J*^ *Prkdc* ^*scid*^ *Il2rg* ^*tm1Wjl*^/WaskJ (NSGW41) mice were a kind gift by Prof. Claudia Waskow.^27^ Mice were bred at the German Cancer Research Center in Heidelberg under pathogen‐free conditions. All animal experiments were performed after approval of the official committee (Regierungspräsidium Karlsruhe) and in accordance with the authorized regulatory guidelines.

### RNA‐sequencing analysis

For RNA‐sequencing (RNA‐seq), cord blood units from eight donors were thawed, pooled, and pre‐stimulated for 48 h, followed by lentiviral transduction in triplicates with high titer lentiviral particles containing shLPIN1.1159, shLPIN1.452, shLPIN1.957, or shLuciferase as control. OCI‐AML3 cells were transduced in triplicates. FACS‐sorted fluorescence‐positive cells were used for RNA extraction. For details, see the Supplemental Methods section.

### Lipid analysis of cells and subcellular fractions

Snap frozen cells were subjected to lipid extractions using an acidic liquid–liquid extraction method,^28^ except for plasmalogens, which were extracted under neutral conditions. Samples were analyzed on QTRAP 6500+/5500 (Sciex, Framingham, Massachusetts, USA) and Q Exactive (Thermo Fisher Scientific, Waltham, Massachusetts, USA) mass spectrometers, with chip‐based (HD‐D ESI Chip, Advion Biosciences, Ithaka, New York, USA) electrospray infusion and ionization via a Triversa Nanomate (Advion Biosciences) as described.^29^ For details on cell preparation, lipid extraction, MS analysis, and data evaluation, see the Supplemental Methods section.

### Statistical analyses

For RNA‐seq and lipidomics analyses, statistical tests are described in the corresponding sections. Assays were analyzed with GraphPad Prism version 8 and beyond. A precise description of the applied tests can be found in the Supplemental Methods section. Asterisks indicate the following *p* value or adjusted *p* value levels if not otherwise specified in the text: *p ≤ 0.05, **p ≤ 0.005, ***p ≤ 0.0005. ns indicates no significant results.

See the Supplemental Methods section for further experimental details and Table S1 for material details.

## RESULTS

### *LPIN1* expression correlates with LSC frequency and prognosis in AML

**Figure 1:**
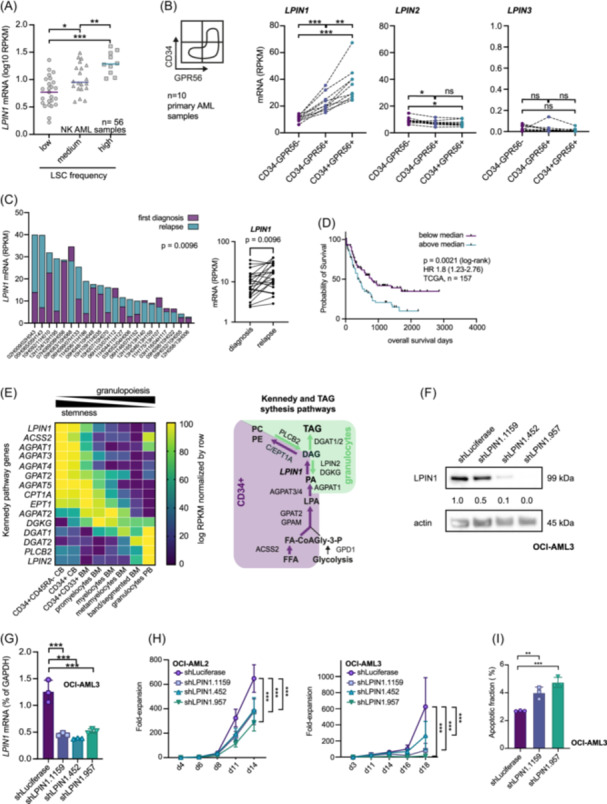
***LPIN1* expression correlates with leukemic stem cell frequency and prognosis in acute myeloid leukemia. (A)** Dot plot of *LPIN1* mRNA expression in NK AML samples (*n* = 56) with low (*n* = 25, round symbol, lilac bar), medium (*n* = 20, upside triangle symbol, blue bar), or high (*n* = 10, square symbol, green bar) LSC frequency. LSC frequencies were determined in immunocompromised mice (NSG) via limiting dilution assays (LSChigh: LSC frequencies > 1 in 32,000; LSClow: LSC frequencies < 1 in 3 × 10⁶; LSCmedium: in between the groups). The data are part of the Leucegene project.^30^ Symbols represent individual samples, horizontal bars show median *LPIN1* expression, and asterisks show results from Mann–Whitney *U* test. **(B)** Left panel: Schematic sketch of the sorting strategy of NK AML samples (*n* = 10); right panel: from left to right *LPIN1*, *LPIN2*, and *LPIN3* mRNA expression in CD34–GPR56– (lilac), CD34–GPR56+ (blue), and CD34+GPR56+ sorted fractions (green). Asterisks show results from a repeated measures one‐way ANOVA with Geisser–Greenhouse correction and Holm‐Sidak's multiple comparison test with individual variances computed for each comparison. **(C)** *LPIN1* mRNA expression in 22 paired primary AML samples from the Leucegene project at initial diagnosis (lilac) and relapse (blue) shown as stacked bar plot (left) and column dot plot (right). Paired *t*‐test. **(D)** Adapted Kaplan–Meyer plot showing overall survival in dependence of *LPIN1* mRNA expression from TCGA PanCancerAtlas.^31^ Survival data with >1 day follow‐up time were available for 157 patients. Patient groups were divided by median *LPIN1* expression. **(E)** Left: Heatmap showing expression of genes of the Kennedy pathway in different maturation stages during myelopoiesis using RNA‐seq data from the Leucegene project. Yellow indicates higher expression values and lilac indicates lower expression values (log10 RPKM). CB: cord blood, BM: bone marrow. Right: Graphic illustrating triacylglycerol and phospholipid synthesis in HSCs (lilac) and differentiated granulocytes (green). Bold letters and arrows indicate higher expression. **(F)** Western blot showing LPIN1 protein expression in OCI‐AML3 upon lentiviral transduction with shRNAs against *LPIN1* or *Luciferase* (shLuc). Actin was used as a loading control. **(G)** Knockdown (KD) efficiency of three different shRNAs against *LPIN1* compared to shLuc in OCI‐AML3 measured by q‐RT‐PCR (shLuc: lilac, shLPIN1.1159: blue, shLPIN1.452: turquoise, shLPIN1.957: green). Three replicates were used per condition. Data are shown as mean + SD. Symbols represent individual replicates. Asterisks show results from ordinary one‐way ANOVA corrected for multiple comparisons with Dunnett's multiple comparison test with a single pooled variance. **(H)** Left: Cell proliferation for OCI‐AML2 transduced with shLPIN1.1159 (blue), shLPIN1.452 (turquoise), or shLPIN1.957 (green) versus shLuc (lilac). Shown is the fold‐increase in absolute cell counts per well until day 14, normalized to the fourth day of the culture. For each condition, 8 replicates were started. Cells were counted by HTS‐FACS. Right: Proliferation of OCI‐AML3 transduced with shLPIN1.1159 (blue), shLPIN1.452 (turquoise), or shLPIN1.957 (green) versus shLuc (lilac). Shown is the fold‐increase in absolute cell counts per well until day 18, normalized to the third day of the culture. For each condition, eight replicates were started. Cells were counted by HTS‐FACS. **(I)** Apoptosis assay for OCI‐AML3 transduced with shLPIN1.1159 (blue) or shLPIN1.957 (green) versus shLuc (lilac). Shown is the fraction of apoptotic cells. Three replicates were used per condition. Data are shown as mean + SD. Symbols represent individual replicates. Asterisks show results from ordinary one‐way ANOVA corrected for multiple comparisons with Dunnett's multiple comparison test with a single pooled variance. DG, diacylglycerol; FA‐CoA, fatty‐acyl‐CoA; Gly3‐P, glycerol‐3‐phosphate; LPA, lyso‐phosphatidic acid; PA, phosphatidic acid; PC, phosphatidylcholine; PE, phosphatidylethanolamine; TG, triacylglycerol.

In a previous screen for novel LSC markers, we observed that the PAP LPIN1 was associated with a high LSC burden.^30^ *LPIN1* mRNA expression positively correlated with LSC frequencies in 56 mostly normal karyotype (NK) AML samples (Figure 1A). When reanalyzing our previous data in more detail with regards to *LPIN1* and its network, we found that the LSC‐enriched CD34+GPR56+ compartment possessed higher *LPIN1* expression compared to the CD34 negative fractions in 10 sorted samples (Figure 1B). The association with the LSC compartment was specific to *LPIN1*, whereas *LPIN2* and *LPIN3* were either similarly expressed across the three fractions or showed no specific expression pattern. Within the intermediate risk group, *LPIN1* had the highest expression levels in AML with co‐mutations in *NPM1*, *FLT3*, and *DNMT3A*, which we had previously reported to possess high LSC frequencies and to be associated with poor outcomes (Figure S1A).^32^ Moreover, *LPIN1* but not *LPIN2* or *LPIN3* expression was significantly increased in paired relapse samples compared to the initial diagnosis (Figures 1C and S1B). In line, only *LPIN1* expression correlated with decreased overall survival of AML patients from the TCGA PanCancerAtlas (Figure 1D and Table S2)^31^ and with decreased event‐free survival from datasets accessed by Kaplan–Meier Plotter^33^, ^34^, ^35^ (Figure S1C).

From experiments in adipocytic tissue, it is known that LPIN1 exerts two distinct functions, as a PAP enzyme in generating DG required for TG and glycerophospholipid synthesis, and as a transcription factor coactivator (Figure S1D).^15^, ^18^, ^20^, ^21^ To approach its function in the context of hematopoiesis, we first analyzed the expression levels of key enzymes participating in TG and glycerophospholipid production using our previously published RNA‐seq data of hematopoietic cells (Figure 1E, left panel). Genes that contributed to glycerophospholipid production including *LPIN1* were upregulated in more immature cells and decreased during myeloid differentiation. In contrast, genes such as *DGAT2*, which are associated with enhanced TG synthesis and reduced glycerophospholipid production, showed increased expression throughout differentiation, reaching their highest levels in terminally differentiated peripheral blood granulocytes. These expression dynamics would be in line with a metabolic switch during myeloid differentiation from glycerophospholipid toward TG production (Figure 1E, right panel).

For functional experiments, we designed three shRNAs against *LPIN1* and a control targeting renilla luciferase (shLuc), and validated the knockdown (KD) efficiency after lentiviral transduction on mRNA and protein levels in several cell lines using Ametrine as a fluorescent marker to monitor the gene transfer (Figures 1F,G and S1E,F). In the first functional assays performed with cell lines, we found that suppression of *LPIN1* resulted in reduced cell proliferation in OCI‐AML2 and OCI‐AML3, but not in the K562 cell line, which has no detectable p53 protein expression (Figures 1H and S1G). As a potential mechanism for reduced cell expansion in the cell lines with functional p53 axis, we observed a slightly higher fraction of apoptotic cells upon *LPIN1* KD (Figure 1I). This might be underestimated by gating on successfully transduced Ametrine‐positive cells. No differences in the distribution of cell cycle phases were observed (Figure S1H). Together, these data pointed toward a functional role of *LPIN1* in leukemia warranting further investigation in primary human samples.

### *LPIN1* maintains the stem cell‐enriched CD34+ compartment and proliferative capacity of AML cells in vitro and in vivo

**Figure 2:**
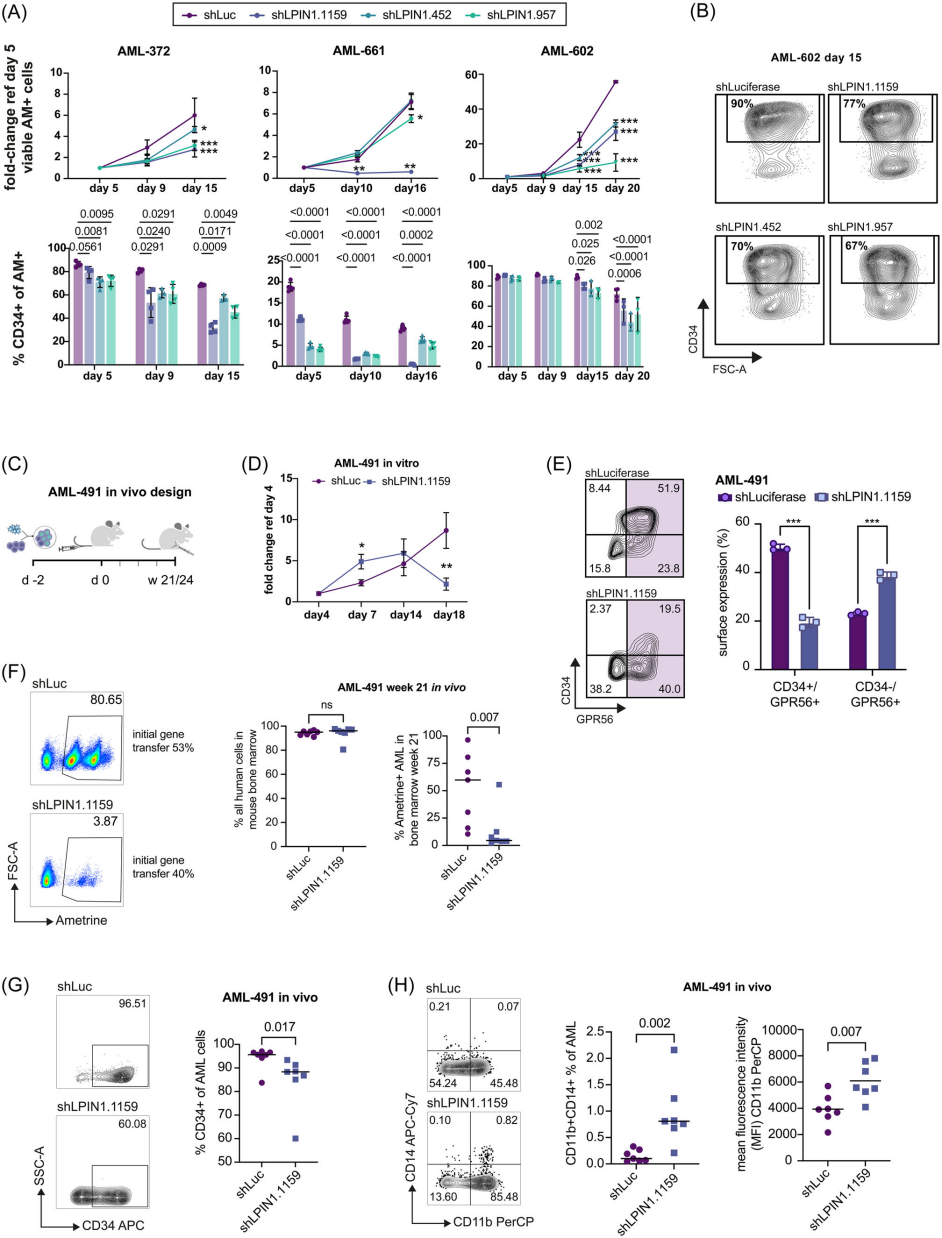
***LPIN1* is required for AML cell proliferation, in vivo engraftment, and maintenance of the stem cell‐enriched CD34+ compartment. (A)** Top: Proliferation of patient‐derived xenograft (PDX) AML cells transduced with shLPIN1.1159 (blue), shLPIN1.452 (turquoise), or shLPIN1.957 (green) versus shLuc (lilac). Shown is the fold‐increase in absolute cell counts per well normalized to day 5. For each condition, five replicates were started. Cells were counted by HTS‐FACS. A two‐way ANOVA was performed, followed by the Holm‐Sidak test to correct for multiple comparisons. Bottom: Bar plots indicating the CD34+ bright fraction of AM+ cells at the respective time points. A two‐way ANOVA was performed, followed by the Holm‐Sidak test to correct for multiple comparisons. **(B)** Representative FACS plots showing CD34 expression and FSC‐A of AML‐602 cells on day 15 after lentiviral transduction with the indicated shRNAs. Values indicate CD34+ bright fractions in percent. **(C)** Graphic illustrating the experimental setup of in vivo experiments. See “Materials and Methods” section for details. For initial gene transfer, see Table S4. Created with BioRender.com. **(D)** Proliferation of primary AML‐491 cells transduced with shLPIN1.1159 (blue) versus shLuc (lilac). Shown is the fold‐increase in absolute cell counts per well normalized to the fourth day of culture. For each condition, five replicates were started. Cells were counted by HTS‐FACS. **(E)** Left: Representative FACS plots showing CD34 and GPR56 expression of primary AML‐491 cells transduced with shLPIN1.1159 (bottom) versus shLuc (top) on day 12. Values indicate gated fractions in percent. Right: Bar plot showing CD34 and GPR56 expression of primary AML‐491 cells in vitro transduced with shLPIN1.1159 (blue) versus shLuc (lilac). For each condition, three replicates were measured. Data are shown as mean + SD. Symbols represent individual replicates. **(F)** Left panel: Representative FACS plots showing FSC‐A and ametrine expression of primary AML‐491 cells transduced with shLPIN1.1159 (bottom) versus shLuc (top) 21 weeks after transplantation into immunocompromised NSGW41 mice. Values indicate gated fractions in percent. Right panel: Dot plots showing the overall human leukemic engraftment (left) and the fraction of Ametrine‐positive cells harboring shRNAs against LPIN1 or control (shLPIN1.1159 versus shLuc) within total bone marrow 21 weeks after injection (right). Human CD45 and CD11b were used to detect overall leukemic engraftment of AML‐491 including non‐transduced Ametrine negative cells, which was similar in both conditions, thus excluding technical issues during injections. Each group consisted of seven mice represented by symbols. Mann–Whitney *U* test. **(G)** Left: Representative FACS plots showing CD34 expression and SSC‐A of primary AML‐491 cells transduced with shLPIN1.1159 (bottom) versus shLuc (top) 21 weeks after transplantation into immunocompromised NSGW41 mice. Values indicate gated fractions in percent. Right: Dot plot showing CD34 surface expression of primary AML‐491 cells transduced with shLPIN1.1159 (blue) versus shLuc (lilac) 21 weeks after transplantation. Each group consisted of seven mice represented by symbols. Mann–Whitney *U* test. **(H)** Left: Representative FACS plots showing CD14 and CD11b expression of primary AML‐491 cells transduced with shLPIN1.1159 (bottom) versus shLuc (top). Values indicate gated fractions in percent. Center: Dot plot showing the frequency of CD14 and CD11b co‐positive fractions of primary AML‐491 cells transduced with shLPIN1.1159 (blue) versus shLuc (lilac). Each group consisted of seven mice, and each symbol represents one mouse. Mann–Whitney *U* test. Right: Dot plot showing the mean intensity of CD11b in primary AML‐491 cells transduced with shLPIN1.1159 (blue) versus shLuc (lilac). Unpaired *t*‐test after passing normal distribution test.

To determine the impact of *LPIN1* silencing on primary AML cells, three patient‐derived xenograft (PDX) AML samples including one with complex karyotype and TP53 mutation (AML‐661, AML‐372, and AML‐602; see Table S3 for genetic details) were transduced with shRNAs against *LPIN1* or *shLuc* control. Leukemic cell expansion was significantly reduced with all three shRNAs in AML‐372 and AML‐602 and with two shRNAs in AML‐661 (Figure 2A). Since *LPIN1* was identified as a potential LSC‐associated gene, we also assessed the expression of the stemness marker CD34. We observed a significant reduction of the CD34+LSC‐enriched compartment in all three samples with all three shRNAs even at time points at which no overall impact on proliferation was seen, suggesting that *LPIN1* KD affected both proliferation and stemness (Figure 2A,B). To better estimate the impact of *LPIN1* KD on leukemia stem cell function we transplanted AML‐491, the matched first diagnosis sample of AML‐661, into two different mouse strains (outlined in Figure 2C). Before transplantation, we confirmed a significant reduction of *LPIN1* mRNA (Figure S2A), cell expansion, and expression of the two LSC‐markers CD34 and GPR56 in AML‐491 with two different *LPIN1* shRNAs in vitro (Figures 2D,E and S2B). Having confirmed the *LPIN1* KD phenotype in this sample we transplanted unsorted PDX cells with similar gene transfer in *LPIN1* KD and control conditions (Table S4), and analyzed leukemic burden in NSGW41 and NSG mouse bone marrow after 21 and 24 weeks, respectively (Figures 2F,G and S2C,D). We observed reduced engraftment of *LPIN1* KD cells (Ametrine positive, AM+) in the two independent experiments (Figure 2F [NSGW41] and Figure S2C [NSG]). Total human leukemia engraftment including transduced AM+ and non‐transduced AM‐negative (AM–) cells determined by human CD11b and CD45 staining was similarly high (median >95%) in NSGW41 mice excluding technical issues (Figure 2F). As observed in the in vitro experiments, the CD34+ population was significantly reduced in the AM+ *shLPIN1* compared to the AM+ *shLuc* mice (Figures 2G and S2D). As another quality control, we confirmed robust *LPIN1* KD levels by qRT PCR after sorting the AM+ cells from engrafted mice (Figure S2E). To test whether the loss of the CD34+ LSC‐enriched compartment was accompanied by differentiation, we assessed myeloid and monocytic differentiation markers CD11b and CD14 on the engrafted cells. Although the number of CD14+ cells was low in the NSGW41 mouse experiment, we observed a significantly higher fraction of CD11b+CD14+ cells within the *LPIN1* KD cells compared to *shLuc* control cells (Figure 2H). When comparing the mean fluorescence intensity (MFI) for CD11b reflecting the number of surface molecules on the cells, the CD11b‐MFI was significantly higher in *LPIN1* KD cells in vivo compared to *shLuc* in both mouse strains (Figures 2H and S2F,G).

Taken together, in vitro and in vivo data established a functional role of *LPIN1* for human AML including the maintenance of the immature LSC pool.

### *LPIN1* is required for in vivo engraftment and lymphoid differentiation potential of cord blood CD34+ cells

**Figure 3:**
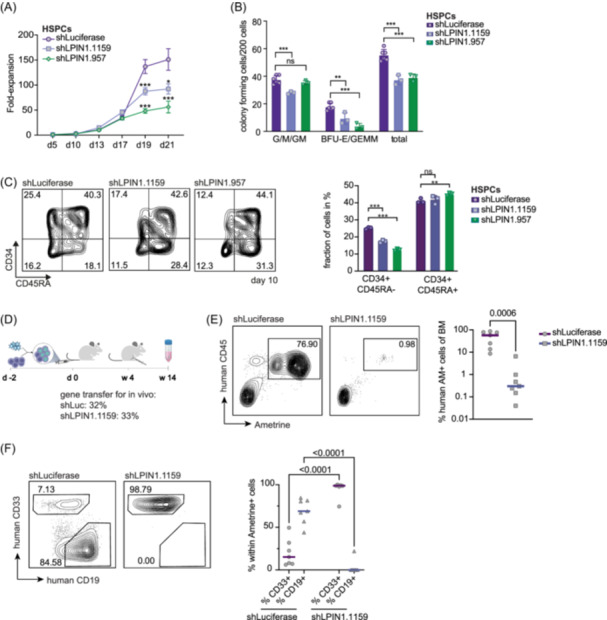
**Normal CD34+ HSPCs are susceptible to** ***LPIN1*** **loss. (A)** Proliferation curves for HSPCs transduced with shLPIN1.1159 (blue) or shLPIN1.957 (green) versus shLuc (lilac). Shown is the fold‐increase in absolute cell counts per well on day 21 normalized to the fifth day of the culture. For each condition, five replicates were started. Cells were counted by HTS‐FACS. **(B)** Colony‐forming cell assay of pooled HSPCs, which were transduced with shLPIN1.1159 (blue) or shLPIN1.957 (green) versus shLuc (lilac) as negative control. Ametrine‐positive cells were directly sorted into methylcellulose 72 h post‐transduction. 200 cells were plated per well and colonies were counted 10 days post‐plating. **(C)** Left: Representative FACS plots showing CD34 and CD45RA expression of HSPCs 10 days after transduction with shLPIN1.1159 (middle) and shLPIN1.957 (right) versus shLuc (left). Values indicate gated fractions in percent. Right: Bar plot of the measured CD34 and CD45RA expression of HSPCs 10 days after transduction with shLPIN1.1159 (blue) or shLPIN1.957 (green) versus shLuc (lilac). For each condition, three replicates were measured. Data are shown as mean + SD. Symbols represent individual replicates. **(D)** Graphic illustrating the experimental setup of in vivo experiments. For initial gene transfer, see Table S4. See “Materials and Methods” section for details. Created with BioRender.com. **(E)** Left: Representative FACS plots showing ametrine and CD45 expression of HSPCs transduced with shLPIN1.1159 (right) versus shLuc (left) 14 weeks after transplantation into immunocompromised mice. Values indicate gated fractions in percent. Right: Dot plot showing engraftment of HSPCs transduced with shLPIN1.1159 (blue) versus shLuc (lilac) 14 weeks after transplantation into immunocompromised mice. Each group consisted of seven mice represented by symbols. Mann–Whitney *U* test. **(F)** Left: Representative FACS plots showing CD19 and CD33 expression in HSPCs transduced with shLPIN1.1159 versus shLuc 14 weeks after transplantation into immunocompromised mice. Values indicate gated fractions in percent. Right: Dot plot showing the fraction of CD19 and CD33 positive output of engrafted HSPCs transduced with shLPIN1.1159 (blue) versus shLuc (lilac) 14 weeks after transplantation into immunocompromised mice. Each group consisted of seven mice represented by symbols. Mann–Whitney *U* test. BFU‐E, burst‐forming unit‐erythroid; CFU, colony‐forming unit; G, granulocyte; GEMM, granulocyte, erythrocyte, megakaryocyte, macrophage; GM, granulocyte/macrophage; M, macrophage.

To further investigate the pronounced impact of *LPIN1* on the CD34+ compartment, we used healthy cord blood (CB) CD34+ cells (HSPCs) for in vitro and in vivo experiments after confirming the KD efficiency of the three shRNAs against *LPIN1* in CB CD34+ by qPCR (Figure S3A). *LPIN1* suppression reduced HSPC expansion (Figures 3A and S3B) and colony‐forming capacity (Figures 3B and S3C). As observed in leukemic cells, *LPIN1* suppression affected the immature multipotent progenitors (CFU‐GEMM) more than committed progenitors (CFU‐G, CFU‐M; Figure 3B). Accordingly, the stem‐cell enriched CD34+CD45RA− fraction was more rapidly lost in vitro by *LPIN1* suppression compared to the CD34+CD45RA+ fraction (Figure 3C). Moreover, the proliferative output of sorted CD34+CD45RA− cells was hampered by both *LPIN1* shRNAs, while the proliferative capacity of sorted CD34+CD45RA+ cells, which are devoid of stem cell activity, was not robustly impaired (Figure S3D).

To understand the role of *LPIN1* for HSC activity *in vivo* we injected lentivirally transduced CB CD34+ cells in immunocompromised NSGW41 mice and observed impaired bone marrow engraftment of human cells at week 14 in mice receiving *shLPIN1* transduced cells (Figure 3D,E). When assessing the differentiation potential of the engrafted HSCs, we found that the very few *LPIN1* KD cells engrafting in mice mostly differentiated into myeloid CD33+ cells, but did not produce CD19+ B lymphoid cells (Figure 3F). Together, the functional in vitro and in vivo experiments revealed that *LPIN1* is required to maintain the proliferative capacity and immature, undifferentiated phenotype of AML and healthy HSPCs.

### LPIN1 *regulates expression of genes for glycerophospholipid metabolism in OCI‐AML3 and HSPCs*

**Figure 4:**
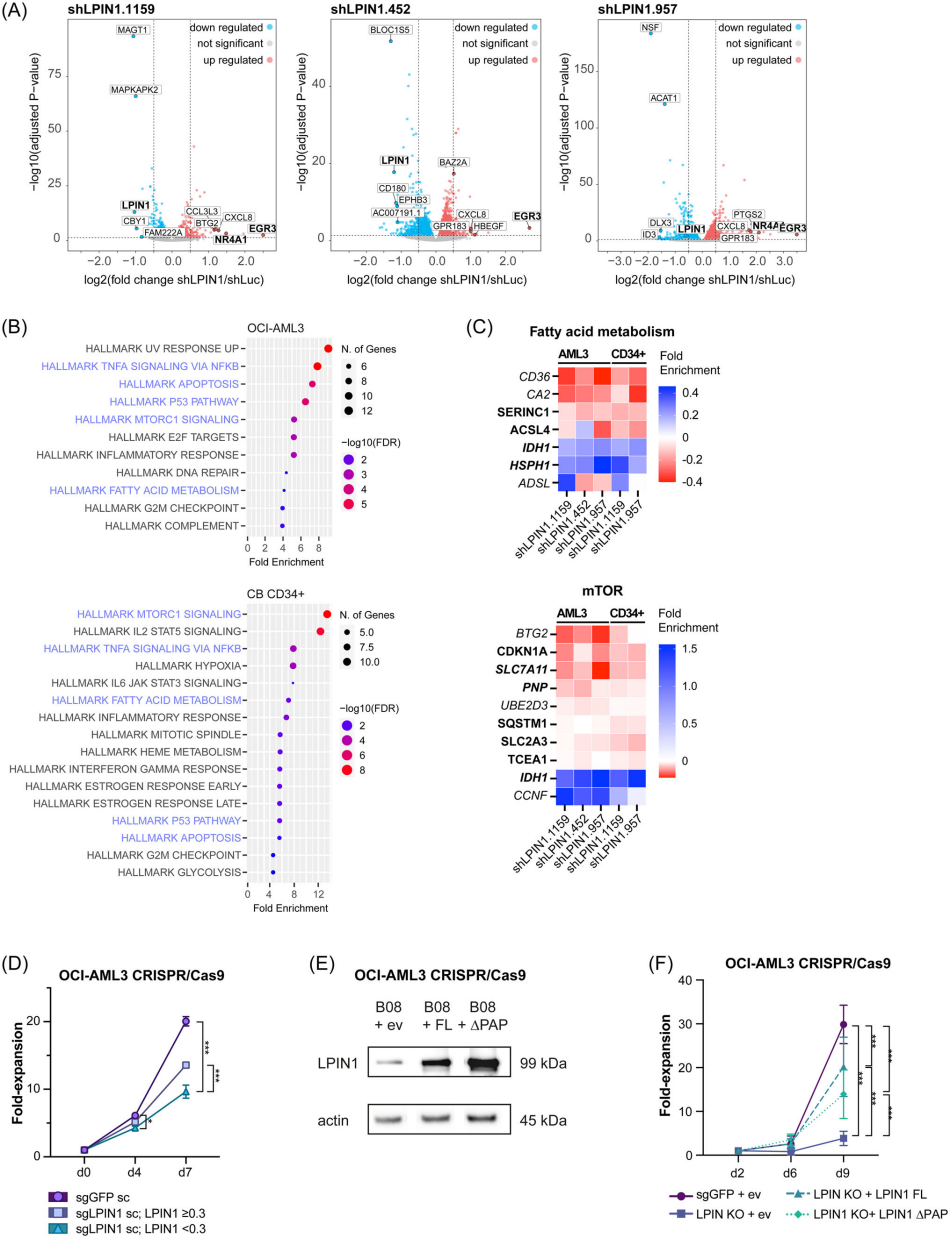
***LPIN1*** **regulates the expression of genes for glycerophospholipid metabolism in OCI‐AML3 and HSPCs. (A)** Volcano plot showing the log2‐fold changes (log2FC) of gene expression (*x*‐axis) and log10‐transformed adjusted *p* values (*y*‐axis) for OCI‐AML3 from RNA‐seq data, 48 h after lentiviral transduction with shLPIN1.1159, shLPIN1.452, or shLPIN1.957 versus shLuc. Data points in gray represent genes without significant regulation. Data points highlighted in blue represent significantly downregulated genes (*p* ≤ 0.05, log2FC shLPIN1/shLuc < 0), while data points highlighted by red dots represent significantly upregulated genes (*p* ≤ 0.05, log2FC shLPIN1/shLuc > 0). Not all gene symbols are displayed due to space constraints. See Table S5 for full gene lists. **(B)** Bubble plot showing the fold enrichment of significantly regulated gene sets in OCI‐AML3 (top) and HSPCs (bottom). Pathway analysis was performed using Shiny GO^36^ and the hallmark.MSigDB database. **(C)** Heatmap illustrating the regulation of genes belonging to the “FATTY ACID METABOLISM” (top) and “MTORC1 SIGNALING” gene set (bottom) in OCI‐AML3 and HSPCs. Bold gene names indicate significant regulation in OCI‐AML3, italic gene names indicate significant regulation in HSPCs, and bold italic gene names indicate significant regulation in both. Fold enrichment is given as log2FC shLPIN1/shLuc. Upregulation is indicated by blue, whereas downregulation is indicated by red color. **(D)** Proliferation of grouped OCI‐AML3 single clones with either equal and more (blue), or less (turquoise) than 30% remaining LPIN1 protein after knockout (KO) using CRISPR/Cas9 compared to sgGFP single clones (lilac) as control. For each sgLPIN1 clone, six replicates were started. For sgGFP, eight single clones at six replicates were started as a reference. Cells were counted by HTS‐FACS. Data are given as mean + SEM. **(E)** Western blot showing *LPIN1* overexpression upon lentiviral transduction of OCI‐AML3 with *LPIN1* full length (FL) (middle), an enzymatically impaired version of *LPIN1* (ΔPAP) (right), or empty vector (ev) (left). Actin was used as a loading control. **(F)** Proliferation of OCI‐AML3 sgLPIN1 single clone 8 (sc #8) after lentiviral transduction with *LPIN1* full length (FL), the enzymatically impaired version of LPIN1 (ΔPAP), or empty vector (ev) compared to a sgGFP single clone (lilac) upon lentiviral transduction with ev as control. Shown is the fold‐increase in absolute cell counts per well normalized to the second day of the culture. For each condition, eight replicates were started. Cells were counted by HTS‐FACS. Data are given as mean + SD. Asterisks show results from two‐way ANOVA with Dunn's multiple comparison test.

To understand the processes LPIN1 acts on, we performed RNA‐seq in HSPCs and OCI‐AML3 upon LPIN1 KD (Figures 4A and S4A). RNA‐seq analysis confirmed a significant reduction of *LPIN1* expression with the used shRNAs (Figure S4B). In addition, LPIN1 KD caused broad, but only subtle changes in gene expression with log2‐fold changes ranging from –0.87 to 1.74 in HSPCs and –1.21 to 3.49 in OCI‐AML3 (Figures 4A and S4C and Table S5). Overall, we identified 199 differentially expressed genes (DEG) in OCI‐AML3 and 88 DEG in HSPCs upon LPIN1 KD (padj. ≤0.05; DeSeq. 2), of which 22 genes overlapped. Gene set enrichment analysis using Shiny GO and the hallmark.MSigDB pathway database (FDR ≤ 0.05; Figure 4B)^36^ revealed significant enrichment for “TNFA SIGNALING VIA NFKB,” “APOPTOSIS,” “P53 PATHWAY,” “MTORC1 SIGNALING,” “FATTY ACID METABOLISM,” “INFLAMMATORY RESPONSE,” and “G2M CHECKPOINT.” The gene sets “MTORC1 SIGNALING” and “FATTY ACID METABOLISM” showed the most consistent changes of DEG between the two cell types (Figure 4C).

The rather subtle changes in gene expression suggested that the enzymatic role of LPIN1 might be driving the phenotype in AML and HSPCs more than its function as a transcription factor coactivator. To differentiate between these, *LPIN1* knockout (KO) single clones were created using clustered regularly interspaced short palindromic repeats/CRISPR associated protein 9 (CRISPR/Cas9) technique (Figure S4D). As expected, OCI‐AML3 single *LPIN1* KO clones showed a disadvantage in proliferation compared with non‐target (GFP) sgRNA controls (Figures 4D and S4E). To determine whether the observed phenotype in OCI‐AML3 single clones could be rescued, we created plasmids encoding either a fully functional LPIN1 protein (LPIN1 FL) or an enzymatically impaired variant (LPIN1 ΔPAP), which we generated by mutating the DXDXT to an EXEXT motif as described by Peterson et al. (Figure S4F).^37^ Overexpression vectors were validated for protein overexpression (Figure 4E) and physiologic intracellular localization (Figure S4G). Overexpression of the LPIN1 ΔPAP was significantly less efficient in rescuing the proliferation disadvantage of the *LPIN1* KO compared to the full‐length version suggesting that the enzymatic function of LPIN1 is crucial for these cells (Figure 4F).

### *LPIN1* regulates phosphatidylcholine (PC) and ‐ethanolamine levels in AML

**Figure 5:**
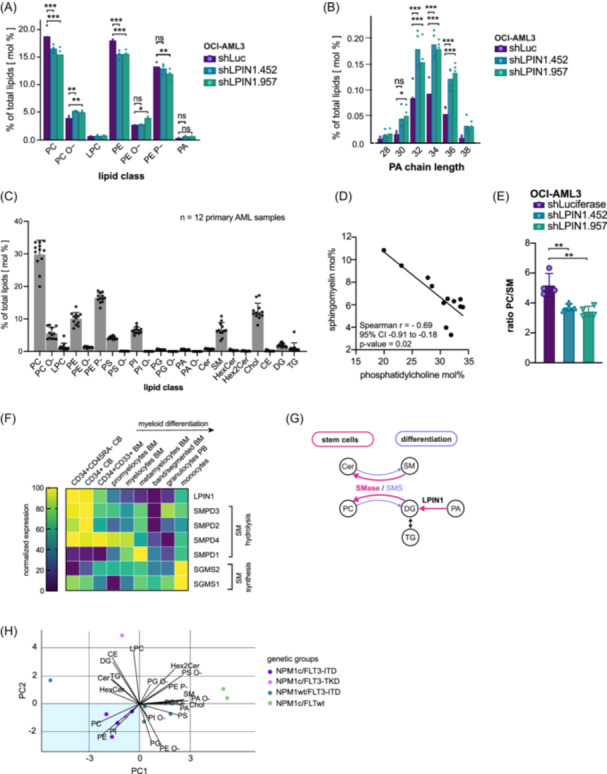
***LPIN1*** **regulates phosphatidylcholine and ‐ethanolamine levels in AML. (A)** Bar plot showing the distribution of lipid classes in OCI‐AML3 after transduction with shLPIN1.452 (turquoise) or shLPIN1.957 (green) versus shLuc (lilac) measured by mass spectrometry. For each condition, four replicates were measured. Data are shown as mean. Symbols represent individual replicates. **(B)** Bar plot showing changes in phospholipid composition regarding PA chain length in OCI‐AML3 after transduction with shLPIN1.452 (turquoise) or shLPIN1.957 (green) versus shLuc (lilac) measured by mass spectrometry. For each condition, four replicates were measured. Data are shown as mean. Symbols represent individual replicates. **(C)** Bar plot showing the lipid composition of primary AML samples measured by mass spectrometry. Each point represents a sample. Data are shown as mean + SD. **(D)** *XY*‐plot displaying the relative abundance of phosphatidylcholine and sphingomyelin in primary AML samples, measured by mass spectrometry, in mol%. Pearson coefficient was calculated and a simple linear regression model using a 95% confidence interval was applied. **(E)** Bar plot showing the ratio of PC/SM in OCI‐AML3 after transduction with shLPIN1.452 (turquoise) or shLPIN1.957 (green) versus shLuc (lilac). Data are shown as mean + SD. Symbols represent individual replicates. Ordinary one‐way ANOVA corrected for multiple comparisons with Dunnett's multiple comparison test with a single pooled variance. **(F)** Heatmap showing gene expression of sphingomyelin synthases and hydrolases as well as *LPIN1* in different maturation stages during myelopoiesis using RNA‐seq data from the Leucegene project. Yellow indicates higher expression values and lilac indicates lower expression values (log10 RPKM). **(G)** Graphic illustrating the connection between phospho‐ and sphingolipid metabolism. Metabolic processes that are assumed to be upregulated in HSCs and LSCs based on RNA‐seq data are indicated in pink, and processes that are assumed to be upregulated in differentiated cells are indicated in lilac. **(H)** Principal component analysis (PCA) plot showing PC1 and PC2 from lipidomic analyses in primary AML. The figure includes loading vectors radiating from the origin, representing the contributions of individual lipids to the principal components. Points represent individual samples. The mutational status of the samples is indicated by color: nucleophosmin 1 (NPM1c) mutation and internal tandem‐repeat of fms‐like tyrosine‐kinase 3 (FLT3‐ITD) are highlighted in purple, NPM1c mutation and mutation of the tyrosine kinase domain (TKD) of FLT3 are shown in pink, no mutation of NPM1 (NPM1wt) and FLT3‐ITD are indicated in turquoise, and NPM1c mutation and no mutation in FLT3 (FLT3wt) are shown in green. The shaded area is characterized by similar lipid composition and comprises all four samples with NPM1c/FLT3‐ITD co‐mutations. BM, bone marrow; CB, cord blood; CE, cholesterol ester; Cer, ceramide; Chol, cholesterol; DG, diacylglycerol; HexCer, hexosyl ceramide; Hex2Cer, di‐hexosyl ceramide; LPC, lysophosphatidylcholine; PA, phosphatidic acid; PA O‐, ether‐linked phosphatidic acid; PC, phosphatidylcholine; PC O‐, ether‐linked phosphatidylcholine; PE, phosphatidylethanolamine; PE O‐, ether‐linked ethanolamine; PE P‐, ethanolamine plasmalogen; PG, phosphatidylglycerol; PG O‐, ether‐linked phosphatidylglycerol; SM, sphingomyelin; TG, triacylglycerol.

Given that the enzymatic role of LPIN1 seemed to be crucial for the phenotype, we next assessed the effect of LPIN1 KD on lipid metabolism of OCI‐AML3 cells (Table S6). KD of *LPIN1* significantly reduced the abundance of PC and phosphatidylethanolamine (PE) while increasing the fraction of the respective ether lipids (Figure 5A). Additionally, sphingomyelin (SM) and TG showed a trend to increase (Figure S5A). PA, the main substrate of LPIN1, was not significantly different when comparing total levels, but we observed a significant increase after *LPIN1* KD when comparing PA with a chain length of 34–36 and one double bond (Figures 5B and S5B). Levels of DG, the main product of LPIN1, were not significantly different. This has been described by other groups and can be explained by the fact that DG is constantly used in multiple different metabolic pathways.^24^ Together, these data suggested that LPIN1 functions not only as a regulator of glycerophospholipid abundance but also of their molecular species composition in hematopoietic cells.

As lipidomics experiments require large numbers of input cells, it was not feasible to follow the same experimental design in primary AML cells as done with OCI‐AML3. To still gain insights into the lipid distribution of primary human AML, we analyzed the lipidome of 12 AML samples (Figure 5C). Similar to the lipid class profile of OCI‐AML3, PC was the most abundant phospholipid in all primary AML samples (Figure 5C and Table S7) in line with its role as a key membrane component accounting for >50% of membrane lipids in eukaryotic cells.^38^ Correlation analyses revealed that PC, PE, and hexosyl ceramide (HexCer) formed a cluster, as their relative abundances positively correlated (Figure S5C). Furthermore, the higher the PC and PE abundance, the lower PC ether (PC O‐), SM, and cholesterol fractions resulting in significant anticorrelation (Figures 5D and S5D). In line, LPIN1 suppression led to a significantly reduced PC/SM ratio in OCI‐AML3 (Figure 5E). These results suggested that PC and SM levels were regulated by interconnected metabolic pathways such as the “SM cycle,”^39^ in which SM synthases (sphingomyelin synthase 1‐2 (SMS1‐2)^40^ use PC and Cer to generate SM and DG, while SM hydrolases (sphingomyelin phosphodiesterase 1‐4 [SMPD1‐4]) promote the opposite reaction. We again consulted the RNA‐seq dataset of healthy hematopoietic cells and found that SM hydrolases *SMPD2‐4* show a similar expression pattern to *LPIN1*, i.e., they are more highly expressed in immature CD34+ cells and decrease upon myeloid differentiation, while the SM synthases are most highly expressed in mature differentiated monocytes (Figure 5F). Together, these observations suggest that the SM cycle is shifted toward PC/PE in immature hematopoietic cells and that LPIN1 might be involved in this process (Figure 5G).

As the primary human AML cohort used for the lipidomics experiment contained only NK AML specimens, we performed a principal component analysis to determine whether defined molecular genetic groups formed clusters. As shown in Figure S1A, NK‐AML with co‐mutations of NPM1 and FLT3‐ITD have significantly higher LPIN1 expression compared to samples with mutations in only one of these two genes. In line, we observed in the PCA that the four NPM1/FLT3‐ITD co‐mutated samples were all located in close proximity in the PCA, while NPM1 mutated AML with wild‐type FLT3 were located in the opposite direction (Figures 5H and Figure S5E and Table S3).

In summary, we show here that *LPIN1* regulates glycerophospholipid metabolism in AML, that PC is the most abundant lipid in AML and anti‐correlates with SM abundance, and that defined genetic groups cluster by lipid composition.

### Choline pathway inhibition has strong anti‐leukemic activity in patient‐derived xenograft cells

**Figure 6:**
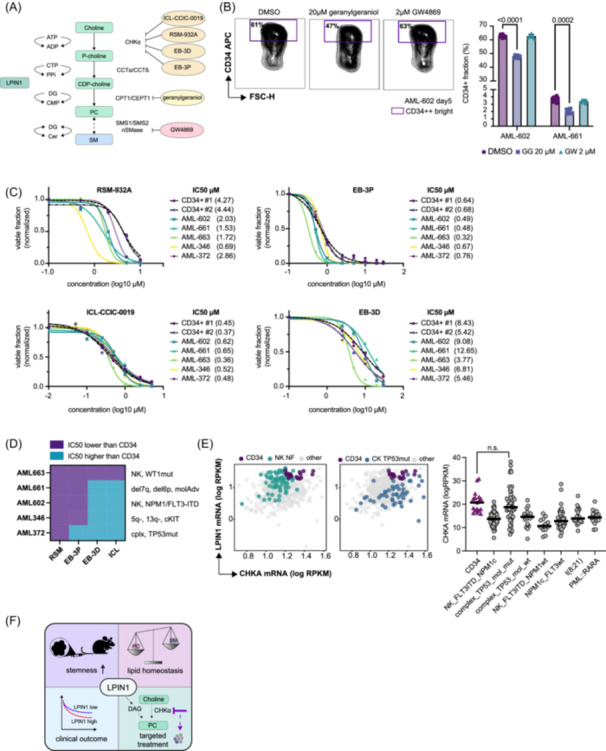
**Choline pathway inhibition has strong anti‐leukemic activity in patient‐derived xenograft cells. (A)** Graphic illustrating the CDP‐choline pathway and the inhibitors used to block key enzymes in human PDX samples. **(B)** Left: Representative FACS plots showing CD34 expression and FSC‐H of AML‐602 cells treated with 20 µM geranylgeraniol (center), 2 µM GW4869 (right), or DMSO as control (left). Values indicate gated fractions of CD34 bright (++) cells in percent. Right: Bar plot showing CD34 surface expression of AML‐602 and AML‐661 cells treated with 20 µM geranylgeraniol (blue), 2 µM GW4869 (green), or DMSO as control (lilac). Data are shown as mean + SD. Symbols represent individual replicates. Numbers indicate results from ordinary two‐way ANOVA and Holm‐Sidak's multiple comparison test with a single pooled variance computed for each comparison. **(C)** Dose–response curves of five PDX samples (AML‐602, AML‐661, AML‐663, AML‐346, and AML‐372) versus two independent batches of healthy HSPCs (CD34+ #1 comprising four donors, CD34 #2 comprising two donors) treated with RSM‐932A, EB‐3P,^41^, ^42^ ICL‐CCIC‐0019, and EB‐3D.^41^, ^42^, ^43^, ^44^ Curves were fitted using a sigmoidal model. Numbers in brackets indicate the half‐maximal inhibitory concentrations (IC50). **(D)** Heatmap comparing the IC50 for the drugs RSM‐932A (RSM), EB‐3P,^41^, ^42^ ICL‐CCIC‐0019 (ICL), and EB‐3D^41^, ^42^, ^43^, ^44^ in AML versus HSPCs (CD34). Lilac indicates a lower IC50 in AML than in HSPCs, blue indicates a higher IC50 in AML than in HSPCs. Annotations indicate the shortened mutational status of the used samples. See Table S3 for further sample information. NK, normal karyotype; *WT1*mut, mutated *wilms tumor protein 1*; del7q, deletion on the long arm of chromosome 7; del6p, deletion on the short arm of chromosome 6; molAdv: adverse risk molecular mutations; *NPM1*: *nucleophosmin*; *FLT3‐ITD*: *fms‐related tyrosine kinase 3* with internal tandem duplication; 5q‐, loss of the long arm of chromosome 5; 13q‐, loss of the long arm of chromosome 13. *cKIT*, *tyrosine kinase KIT*; cplx, complex karyotype; *TP53*mut, mutation of *tumor protein 53*. **(E)** Left: XY plot showing expression of *choline kinase ɑ* (*CHKA*) versus *LPIN1* (log10 RPKM) in samples from the Leucegene cohort. Highlighted are HSPCs (CD34, lilac), normal karyotype samples with *NPM1* and *FLT3*‐ITD co‐mutation (NK NF, green), and complex karyotype samples with *TP53* mutation (CK *TP53*mut, blue). Right: Dot plot of *CHKA* mRNA expression in samples from the Leuegene cohort (log10 RPKM). Symbols represent individual samples, bars show median *CHKA* expression. Statistical significance was tested using ordinary one‐way ANOVA corrected for multiple comparisons with Holm‐Sidak test. CD34: healthy HSPCs; NK_*FLT3ITD*_*NPM1c*: normal karyotype samples with *NPM1* and *FLT3*‐ITD mutation*; complex_TP53_mol_mut:* complex karyotype samples with *TP53* mutation*; complex_TP53_mol_wt:* complex karyotype samples without *TP53* mutation*;* NK_*FLT3ITD*_*NPM1*wt: normal karyotype samples with *FLT3‐ITD* and *NPM1* wild type; *NPM1c*_*FLT3wt*: samples with *NPM1* mutation without *FLT3‐ITD*, t(8;21): translocation of a part from chromosome 8 to chromosome 21; *PML*::*RARA*: reciprocal translocation of *retinoic acid receptor ɑ* (*RARA*) with *promyelocytic leukemia protein (PML)*. **(F)** Graphic illustrating the proposed functions of *LPIN1* in AML and HSPCs. ADP, adenosine diphosphate; ATP, adenosine triphosphate; CCTɑ/CCTβ, choline‐phosphate cytidylyltransferase‐ɑ/choline‐phosphate cytidylyltransferase‐β; CDP‐choline, cytidine‐diphosphate‐choline; Cer, ceramide; CHKɑ1/CHKß, choline kinase‐ɑ1/choline kinase‐β; CMP, cytidine monophosphate; CTP, cytidine triphosphate; nSMase, neutral sphingomyelinase; PC, phosphatidylcholine; P‐choline, phosphocholine; PPi, inorganic phosphate; SM, sphingomyelin; SMS1/SMS2, sphingomyelin synthase 1/sphingomyelin synthase 2.

As lipidomics experiments showed a reduction of PC and PE following *LPIN1* suppression and an anti‐correlation between PC/PE and SM levels, we inhibited key enzymes of the choline pathway, choline kinase alpha (ChoKɑ1), and choline/ethanolamine phosphotransferase 1 (CEPT1), as well as neutral sphingomyelinase (nSMase) in PDX‐AML and healthy CB CD34+ cells using chemicals (outlined in Figure 6A). We expected to see a phenocopy of *LPIN1* KD with the ChoKɑ1 and CEPT1 inhibitors, but opposite effects with the nSMase inhibitor. With the CEPT1 inhibitor geranylgeraniol (GG) and the nSMase inhibitor GW4869 (GW) we did not reach half‐maximal inhibitory concentrations (IC50), even at concentrations of 60 µM (GG) and 5 µM (GW) neither in the five PDX samples nor in healthy CD34+ cells (Figure S6A). However, we noticed a significant reduction of the CD34+ fraction with the CEPT1 inhibitor in AML‐661 and AML‐602 similar to what we observed after *LPIN1* suppression (Figure 6B). The ChoKɑ1 inhibitors were active in reducing the proliferation of primary human AML and CB CD34+ cells at different IC50 ranging from nanomolar concentrations with ICL‐CCIC‐0019 and the non‐commercial compound EB‐3P^41^, ^42^ to lower and higher micromolar concentrations with RSM‐932A and the non‐commercial compound EB‐3D^41^, ^42^, ^43^, ^44^ (Figure 6C). Given that *LPIN1* suppression affected also healthy CD34+ cells, we expected to see activity in healthy cells as well but specifically searched for a compound with a potential therapeutic window. Interestingly, the hill slopes were steeper in PDX cells compared to healthy cells with most drugs (Figure 6C), which might point toward higher dependence on ChoKɑ1 and potentially additional targets in AML cells. RSM‐932A was different from the three other ChoKɑ1 inhibitors, as we detected lower IC50s in all five AML samples compared to the two pools of 2–4 CD34+ donors (Figure 6D). We next investigated a potential association between sensitivity of the AML samples to the drugs and their genetic profiles and found that the most resistant sample AML‐372 had a complex karyotype with biallelic inactivation of TP53 (loss of Chr17 and a somatic mutation in the remaining allele; see Table S3 for genetic information). To further corroborate this observation, we compared *CHKA* and *LPIN1* mRNA co‐expression in the Leucegene AML cohort and found that indeed complex karyotype AML with TP53 mutation, but not with TP53 wild type, expressed the highest *CHKA* levels reaching the high levels detected in CB CD34+ cells (Figure 6E). NK AML with mutations in FLT3‐ITD and NPM1 (NK NF) expressed the highest levels of *LPIN1* compared to all other groups reaching levels of healthy CB CD34+ cells (Figure 6E and Figure S6B), but showed lower *CHKA* expression than complex TP53mut or CB CD34+ (Figure 6E). Good‐risk AML including t(8;21) and PML‐RARA showed low expression of both genes (Figure S6C). Together, these observations establish high co‐expression of *LPIN1* and *CHKA*, two enzymes required for PC/PE production, as a hallmark of immature HSPCs, which is best mimicked by non‐favorable AML. Suppression of either *LPIN1* by genetic knockdown or ChoKɑ1 by small molecule inhibitors suppressed leukemic expansion and might thus offer novel therapeutic approaches in AML (Figure 6F).

## DISCUSSION

Our data provide novel insights into the role of the PAP LPIN1 in the hematopoietic system and AML. We show that *LPIN1* stands out from its related genes *LPIN2* and *LPIN3* by its overexpression in the CD34+ stem cell‐enriched compartment in both AML and normal hematopoiesis, in which it gradually decreases with differentiation. The exclusive expression of LPIN1 in the stem cell compartment might therefore be essential for maintaining phospholipid synthesis in stem‐like cells. Furthermore, we establish that LPIN1 is functionally required to maintain the immature CD34+ compartment. Through knockdown and lipidomics experiments, we provide evidence that LPIN1 as a key producer of DG influences glycerophospholipid and PA levels in AML.

While LPIN1 is required for the maintenance of stemness and phospholipid production in AML, it was shown to be dispensable for mature macrophages, in which it controls the composition of TGs and their incorporation into lipid droplets.^45^ This raises the question of how LPIN1 by producing DG is able to influence diverse metabolic branches in different cell types. Through RNA‐seq data from healthy blood cells, we show here that co‐expression patterns of enzymes driving glycerophospholipid, TG, and SM turnover strongly differ between immature CD34+ and differentiated granulocytes or monocytes, e.g. enzymes of the Kennedy pathway^46^ are overexpressed in CD34+ cells explaining how DG production by LPIN1 serves PC and PE production in these cells. In contrast, enzymes that shift the pathway toward TG production, such as DGAT1^47^ and DGAT2,^48^ are downregulated in CD34+ HSPCs and upregulated in mature granulocytes. LPIN2, previously demonstrated to perform functions distinct from LPIN1 during adipocytic differentiation,^49^ exhibits a similar pattern like DGAT2, suggesting that LPIN2‐derived DG is relevant for TG synthesis in these primary cells. However, LPIN1 being a regulator of glycerophospholipid metabolism does not only influence the overall abundance of these lipids but also the acyl chain lengths and number of double bonds. These two features of lipids have a broad influence on membrane properties including membrane fluidity.^38^

Since lipid metabolism is an intertwined network,^50^ cells can branch off metabolic intermediates from other pathways if synthesis of key metabolites is blocked, or compensate for substrate shortages via upregulation of genes contributing to the same pathway. Interestingly, in OCI‐AML3 the reduction of PC and PE caused by *LPIN1* KD was accompanied by a trend toward increased sphingolipids. In line, PC/PE was anticorrelated with SM in primary AML in our study, and anticorrelation of PC and SM was also shown in other cancer entities, such as non‐small cell lung cancer.^51^ While sphingolipid metabolism was shown to be dysregulated in AML characterized by a shift from anti‐ toward pro‐apoptotic sphingolipids,^52^, ^53^, ^54^, ^55^ the role of glycerophospholipids remains poorly understood. Most data about glycerophospholipid metabolism are derived from experiments with solid tumors, where choline metabolism is frequently upregulated as demonstrated in prostate, lung, and breast cancer.^56^, ^57^ These tumors with a so‐called “cholinic phenotype”^56^ are hypothesized to profit from the upregulation of this pathway by the availability of membrane lipids required for high proliferative rates.^38^, ^56^ Seneviratne et al. were the first to propose that glycerophospholipids like phosphatidylserine modulated stemness and differentiation of AML via regulation of toll‐like‐receptor (TLR) signaling.^58^ Recently, Lo Presti et al. performed an untargeted metabolomics approach on 54 de novo AML patient samples and found a negative impact of phospholipid deregulation on AML prognosis, leading them to suggest PC and PE as biomarkers for aggressive disease.^59^ We show here that NK AML with synergistic co‐occurrence of *NPM1* and *FLT3*‐ITD mutations does not only have the highest *LPIN1* mRNA expression compared to other genetic AML groups but also clusters by a specific phospholipid pattern characterized by high PC/PE and low SM content. We also establish high co‐expression of *CHKA* and *LPIN1* as a hallmark of HSPCs, which is also found in non‐favorable AML groups, but not in good‐risk AML. While complex karyotype AML with TP53 mutation is not particularly high in *LPIN1* expression, we show that it is the only AML group reaching *CHKA* mRNA levels comparable to healthy CB CD34+ cells.

Moreover, we show here that inhibition of CEPT1, which promotes the conversion of CDP‐choline to PC, reduces the CD34+ fraction similar to LPIN1 inhibition, but does not affect overall viability and cell counts, while ChoKɑ1 inhibitors showed strong anti‐proliferative activity on all cells. The reason for this might be that ChoKɑ1 is considered the rate‐limiting enzyme for the first step in PC production,^60^ while the function of CEPT1 might be compensated by other enzymes. Another reason might be that additional off‐targets essential for AML cells are hit by ChoKɑ1, but not CEPT1 inhibitors.

The lower expression of *CHKA* in most AML samples compared to healthy cells together with the lower IC50s of choline kinase inhibitors in AML might point toward a potential therapeutic window. In solid cancers, ChoKɑ1 inhibitors have demonstrated anti‐cancer activity,^61^, ^62^ and TCD‐717 (RSM‐932A) even reached evaluation in a clinical trial (NCT01215864). The distinct anti‐leukemic profiles of various ChoKɑ1 inhibitors shown here highlight the potential of ChoKɑ1 inhibitors in AML and warrant further preclinical investigations in AML. By linking high *LPIN1* expression in AML to high LSC frequency and PC production, we accumulate evidence for the existence of a cholinic phenotype in AML associated with poor prognosis. The high requirement of membrane synthesis is a shared feature between proliferating stem and cancer cells providing a potential rationale for the preference toward phospholipid metabolism in these conditions.^63^

In summary, we establish LPIN1 as a functional regulator of the immature, undifferentiated CD34+ compartment, leukemic cell expansion, and glycerophospholipid levels in AML.

## AUTHOR CONTRIBUTIONS

Karin Huber and Swati Garg performed experiments, analyzed data, generated figures, and wrote the manuscript. Lixiazi He performed in vivo experiments and edited the manuscript. Rui Wang and Lena Schlautmann performed drug profiling and growth curves with primary AML cells. PDX‐AML samples were provided by Irmela Jeremias, who also guided the PDX experiments. Pilar M. Luque‐Navarro and Luisa López‐Cara provided compounds for drug profiling and edited the manuscript. Richard Huth analyzed survival data. Simon Raffel and Alireza Pouya supported experiments and wrote the manuscript. Britta Brügger and Christian Lüchtenborg carried out mass spectrometry measurements, performed computational analyses, and wrote the manuscript. Maike Janssen provided compounds and edited the manuscript. Judith B. Zaugg, Christian Arnold, Carsten Müller‐Tidow, and Christian Rohde performed computational analysis of RNA‐seq data and edited the manuscript. Caroline Pabst led the project and wrote the manuscript.

## CONFLICT OF INTEREST STATEMENT

The authors declare no conflicts of interest.

## ETHICS STATEMENT

Patient samples and cord blood units were collected upon receipt of written informed consent in accordance with the Declaration of Helsinki. The project was approved by the Research Ethics Board of the Medical Faculty of Heidelberg University.

## FUNDING

This work was supported by the FAZIT‐Stiftung. Funding was provided by Deutsche Forschungsgemeinschaft (DFG PA2815/1‐1) and a Max‐Eder‐Grant of the German Cancer Aid to C. Pabst (70114435). This research was also funded by the Consejería de Universidad, Investigación e Innovación of the Junta de Andalucía and FEDER (B‐CTS‐216‐UGR20), the Spanish Ministry of Economy and Competitiveness (PID2019‐109294RB‐I00), and Data storage service SDS@hd supported by the Ministry of Science, Research and the Arts Baden‐Württemberg (MWK) and the German Research Foundation (DFG) through grant (INST 35/1314‐1 FUGG and INST 35/1503‐1 FUGG).

## Supporting information

Supporting information.

Supporting information.

Supporting information.

Supporting information.

Supporting information.

Supporting information.

Supporting information.

Supporting information.

Supporting information.

Supporting information.

Supporting information.

Supporting information.

Supporting information.

Supporting information.

Supporting information.

Supporting information.

Supporting information.

Supporting information.

Supporting information.

Supporting information.

Supporting information.

Supporting information.

## Data Availability

Public RNA‐seq data of the Leucegene project was retrieved from www.leucegene.ca. RNA‐seq data of *LPIN1* KD cells have been deposited in NCBI's Gene Expression Omnibus (59) and are accessible through GEO Series accession number GSE226943. Results from lipidomics analyses of OCI‐AML3 and primary AML are included in the Tables S3 and S5.
