# Supplementary material for: Phosphatidic acid phosphatase LPIN1 in phospholipid metabolism and stemness in hematopoiesis and AML
Source: Hemasphere. 2025 Apr 22;9(4):e70118. doi: 10.1002/hem3.70118 (PMC12012646; doi:10.1002/hem3.70118)
Supplement: Supplementary file 1 — Supporting information. [file HEM3-9-e70118-s005.docx]

# **Supplementa**l **Methods**

## Analysis of the Leucegene and TCGA cohorts

Data from the Leucegene project was analyzed for expression of *LPIN1*, its paralogs *LPIN2* and *LPIN3*, *CHKA*, and other genes involved in glycerophospholipid metabolism. Particularly, differential expression of *LPIN1* and *CHKA* across groups defined by clinical and diagnostic parameters such as prognostic subgroups and mutational status were assessed. For statistical analyses, ordinary one-way ANOVA corrected for multiple comparisons with either Tukey’s or Holm-Sidak multiple comparison tests were applied. The particular tests applied are given in the respective figure legends.

Multivariate survival analysis was performed on data from the TCGA cohort [^1^](https://sciwheel.com/work/citation?ids=8183995&pre=&suf=&sa=0) using *R* version 4.3.3 and the packages *survival*: 3.7-0, *survminer*: 0.4.9, *survivalAnalysis*: 0.3.0, *ggplot2*: 3.5.1, *dplyr*: 1.1.4 and *tidyr*: 1.3.1.

## **RNA-Sequencing analysis**

RNA was extracted from FACS-sorted cells two days after lentiviral transduction using a combination of Trizol and column purification using the RNAeasy mini kit (Qiagen, Hilden, Germany). Before library preparation, RNA quantity and quality were measured by NanoDrop One^C^ Microvolume UV-Vis Spectrophotometer (Thermo Fisher Scientific, Waltham, Massachusetts, USA) and Bioanalyzer 2100 Expert software (Agilent Technologies, Santa Clara, California, USA) (RIN between 9.8-1.0). Eligibility for library preparation was additionally confirmed by test amplification of a 566 bp long GAPDH fragment. Libraries were constructed at EMBL Heidelberg using the TruSeq Stranded Total RNA Library Prep Kit (Illumina, San Diego, California, USA) per manufacturer instructions, SE150 sequencing was performed on a NextSeq 500 Illumina Sequencer (Illumina).

For processing of RNA-sequencing (RNA-seq) data, an in-house designed pipeline using the workflow manager Snakemake for the processing and quality control (QC) of the single-end RNA-seq data was applied [^2^](https://sciwheel.com/work/citation?ids=10318268&pre=&suf=&sa=0). First FastQC 0.11.8 was applied for initial QC, followed by adapter trimming with cutadapt 1.18 using the parameters -m 30, followed by another round of FastQC [^3,4^](https://sciwheel.com/work/citation?ids=15884640,827836&pre=&pre=&suf=&suf=&sa=0,0). Overall, no anomalies or signals indicating data quality issues were detected. Then the samples were aligned to hg38 using STAR and gene counts were quantified with the parameters --alignSJoverhangMin 8 --alignSJDBoverhangMin 1 --alignMatesGapMax 1 000 000 --alignIntronMin 20 --alignIntronMax 1 000 000 --outFilterType BySJout --outFilterMultimapNmax 20 --outFilterMismatchNmax 999 --outFilterMismatchNoverReadLmax 0.04 using the Gencode v28 annotation [^5,6^](https://sciwheel.com/work/citation?ids=49324,43579&pre=&pre=&suf=&suf=&sa=0,0). The whole processing pipeline was summarized with MultiQC 1.6 [^7^](https://sciwheel.com/work/citation?ids=2919136&pre=&suf=&sa=0). Then featureCounts v1.6.3 from the Subread package was used with the parameters -Q 10 -F GTF -g gene_id -t exon -s 2 --ignoreDup and the same Gencode annotation to generate raw read counts for all genes across samples. Finally, a standard DESeq2 workflow with subsequent QC with the design formula "∼ condition" (here: control versus knockdown) based on the raw counts was applied separately for each cell type (OCI-AML3 and CD34^+^ cord blood cells) to obtain raw and adjusted p-values for each gene as well as log2 fold-change estimates [^8,9^](https://sciwheel.com/work/citation?ids=148598,129353&pre=&pre=&suf=&suf=&sa=0,0). The shrunken log2 fold-changes were used as implemented in lfcShrink from DESeq2 with the apeglm method [^10^](https://sciwheel.com/work/citation?ids=43637&pre=&suf=&sa=0).

To perform pathway analysis, gene lists containing genes with significant changes (adjusted p-value ≤ 0.05) were created for each cell type. These were then employed as input for ShinyGO [^11^](https://sciwheel.com/work/citation?ids=8106072&pre=&suf=&sa=0) analysis, using the MSigDB.Hallmark database.

## **Lipid analysis of cells and subcellular fractions**

OCI-AML3 transduced with shRNA against *LPIN1* or *Luciferase* were sorted and expanded. 4x10^6^ cells per OCI-AML3 technical replicate and 1-12x10^6^ per primary AML sample were dissolved in 155 mM ammonium bicarbonate buffer and methanol (1:1, v:v) using sonication (Supp. Table 8-10). Lipid extractions were performed in the presence of internal lipid standards using an acidic liquid-liquid Bligh& Dyer (ABD) extraction method with chloroform:methanol: HCl (20:40:0.3, vol:vol) [^12^](https://sciwheel.com/work/citation?ids=9860151&pre=&suf=&sa=0), except for acid-labile plasmalogens, which were extracted under neutral conditions, omitting HCl. Controls included blank extractions and extractions with internal lipid standards without samples. As a quality control measure, standards were titrated into complex lipid mixtures of known composition. Test ABD extractions were performed where the concentration of PC as a bulk membrane lipid was determined. Based on these test extractions, the sample volumes were adjusted so that the total lipid amounts of the different biological samples are similar. On average, a total lipid amount of 3 000 pmol was used for extractions. Lipid extractions were performed with internal lipid standards for each lipid class, with standards resembling the structure of endogenous lipid species. Following this approach, a relative quantification of lipid species was performed.

Glass Pyrex tubes (16x100 mm) and screw caps with PTFE-liner (Corning, Glendale, Arizona, USA) were used for extractions. Glass pipettes and glass syringes were used for handling of samples in organic solvents. Lipid standards were added from stock solutions (1-10 µM, in chloroform) prior to extractions, using a master mix consisting of 50 pmol phosphatidylcholine (PC, 14:1/14:1, 20:1/20:1; 22:1/22:1, Avanti Polar Lipids, Alabaster, Alabama, USA), 50 pmol sphingomyelin (SM, d18:1/13:0, d18:1/17:0, d18:1/25:0, semi-synthesized [^12^](https://sciwheel.com/work/citation?ids=9860151&pre=&suf=&sa=0), 100 pmol deuterated cholesterol (D7-cholesterol, Avanti Polar Lipids), 28 pmol phosphatidylinositol (PI, 17:0/20:4, Avanti Polar Lipids), 25 pmol phosphatidylethanolamine (PE) and 25 pmol phosphatidylserine (PS) (both 14:1/14:1, 20:1/20:1, 22:1/22:1, semi-synthesized) [^12^](https://sciwheel.com/work/citation?ids=9860151&pre=&suf=&sa=0), 25 pmol diacylglycerol (DG, 17:0/17:0, Avanti Polar Lipids), 25 pmol cholesteryl ester (CE, 19:0, Avanti Polar Lipids, 9:0, 24:1, Sigma-Aldrich, St. Louis, Missouri, USA), and 24 pmol triacylglycerol (TG, D5-mixture, LM-6 000, Avanti Polar Lipids), 5 pmol ceramide (Cer, d18:1/18:0-D3, Cayman Chemical, Ann Arbor, Michigan, USA) and 5 pmol glucosylceramide (HexCer) (GlcCer d18:1/17:0, Avanti Polar Lipids), 5 pmol lactosylceramide (Hex2Cer, d18:1/17:0; Avanti Polar Lipids), 10 pmol phosphatidic acid (PA, 17:0/20:4, Avanti Polar Lipids), 10 pmol phosphatidylglycerol (PG, 14:1/14:1, 20:1/20:1, 22:1/22:1, semi-synthesized) [^12^](https://sciwheel.com/work/citation?ids=9860151&pre=&suf=&sa=0), and 5 pmol lysophosphatidylcholine (LPC, 17:1, Avanti Polar Lipids). The phosphatidylethanolamine plasmalogen (PE P-) standard mix consisted of 22 pmol PE P-Mix 1 (16:0p/15:0-d3, 16:0p/19:0), 31 pmol PE P-Mix 2 (18:0p/15:0-d3, 18:0p/19:0), and 43 pmol PE P-Mix 3 (18:1p/15:0-d3, 18:1p/19:0). Semi-synthesis of PE P- was performed as described [^13^](https://sciwheel.com/work/citation?ids=13678198&pre=&suf=&sa=0) using deuterated fatty acids. The final chloroform phase was evaporated under a gentle stream of nitrogen at 37 °C. Samples were either directly subjected to mass spectrometric analysis or stored at -20 °C prior to analysis, which was typically done within 1-2 days after extraction. Lipid extracts were resuspended in 10 mM ammonium acetate in 60 µl methanol.

Samples were analyzed on a QTRAP6500+ (Sciex, Framingham, Massachusetts, USA) mass spectrometer, except for the measurement of cholesterol acetate, which was done on a QTRAP5500 (Sciex), and TG and DG measurements, which were performed on a Q Exactive (Thermo Fisher Scientific) for OCI-AML3 cells. All MS systems were coupled to a robotic with chip-based (HD-D ESI Chip, Advion Biosciences, Ithaka, New York, USA) electrospray infusion and ionization done via a Triversa Nanomate (Advion Biosciences). For QTRAP6500+ and 5500 (Sciex) measurements, 2 µl aliquots of the resuspended lipid extracts were diluted 1:10 in 10 mM ammonium acetate in methanol in 96-well plates (Eppendorf, Hamburg, Germany). For TG and DG measurements on a Q Exactive (Thermo Fisher Scientific), 5 µl aliquots of the reconstituted lipid extracts were diluted with 15 µl 7.5 mM ammonium formate in isopropanol:methanol (4:2:1, v:v). For cholesterol determinations, the remaining lipid extract was again evaporated and 50 µl acetylchloride:chloroform (1:5, v:v) was added to the tubes. Samples were incubated for 60 min at RT in the open tubes, then the samples were dissolved in 60 µl 10 mM ammonium acetate in methanol and subjected to MS/MS analysis. All MS settings and scan procedures are listed in Supplemental Tables 11-13. Data evaluation was done using LipidView 1.3 beta (Sciex) for QTRAP 6500+/5500 (Sciex) data or LipidXplorer 1.2.8.1 and Peakstrainer for Q Exactive (Thermo Fisher Scientific) data, and in-house-developed software (ShinyLipids and ShinyLipidcountr_alpha4). The amount for endogenous molecular lipid species was calculated based on the intensities of the internal lipid standards.

## **Xenotransplantation**

Thawed HSPCs or freshly harvested PDX AML-491 cells were pre-stimulated overnight in optimal media (see section Cell culture for media components). Cells were transduced with shLuc or shLPIN1.1159 and shLPIN1.452, respectively. After 48 hours, equal numbers of total unsorted cells (fluorescence/-) cells were injected through the tail vein into non irradiated immunocompromised NSGW41 mice (for HSPCs and first AML-491 experiment with shLPIN1.1159), or NSG (second AML491 experiment with shLPIN1.452) mice within 6 hours after sublethal irradiation with 1.75 Gy. Gene transfer of HSPCs was assessed at the second day after infection prior to injection and was 32% in shLuciferase and 33% in shLPIN1.1159 transduced cells. For AML-491 cells gene transfer was measured 4 days after infection post injection using leftover cells. Transduction efficiency was 52.7% for shLuciferase and 40.2% for shLPIN1.1159, and 90.4% for shLuciferase and and 87.1% shLPIN1.452, respectively. Engraftment and surface marker expression were assessed *via* intra-femoral bone marrow aspiration at 3-4 weeks intervals. At the time of final sacrifices cells were harvested from tibia, femur, pelvis and spine for analysis.

Bones were processed as follows: Cells were obtained by careful crushing of the bones in IMDM (Gibco, Waltham, Massachusetts, USA) substituted with 3% FBS (Sigma-Aldrich) and 100 µg/ml DNAseI (Sigma-Aldrich), and tissue and cell aggregates were removed by filtration through a 30 micron filter. Red blood cells (RBC) were removed by lysis with ammonium chloride RBC lysis solution. Cells were washed with PBS and subjected to further analysis *via* flow cytometry and, conserved as cell pellets for qRT-PCR, western blots or frozen in cryotubes to be used later on.

## **Cell culture**

HSPCs were isolated from cord blood units after treatment with DNaseI (Sigma-Aldrich) followed by isolation of mononuclear cells using density gradient centrifugation with PBMC Spin Media (PluriSelect, Leipzig, Germany). HSPCs were further enriched using immuno-magnetic column purification with CD34 MicroBeads (Miltenyi Biotec, Bergisch Gladbach, Germany); purity was measured *via* flow cytometry after staining with CD34-antibody (BD Pharmingen, Franklin Lakes, New Jersey). Cells were either cryopreserved or cultured directly in CD34 media containing IMDM (Gibco) supplemented with 20% BIT (StemCell Technologies, Vancouver, British Columbia, Canada), 100 ng/mL SCF (Miltenyi Biotec), 100 ng/mL FLT3L (Miltenyi Biotec), 50 ng/mL TPO (Miltenyi Biotec), 10-4 M β-mercaptoethanol (Gibco) and 50 µg/mL Gentamicin (Gibco). UM171 (StemCell Technologies) was added freshly to the media at a final concentration of 35nM.

Cryopreserved samples were thawed using melting media containing IMDM (Gibco) with 20% FBS (Sigma-Aldrich) and 100 µg/mL DNase I (Sigma-Aldrich). Frozen cryo vials were thawed in a 37°C water bath, cells were transferred into 50mL tubes, prewarmed melting media was added dropwise to the walls of the tubes under continuous gentle shaking. The tubes were centrifuged at 1 000 rpm for 10 minutes without breaks. Depending on the cell type, cell pellets were resuspended in the respective medium. Primary AML samples were cultured in IMDM (Gibco) supplemented with 15% BIT (StemCell Technologies), 100ng/mL SCF (Miltenyi Biotec), 50ng/mL FLT3L (Miltenyi Biotec), 20ng/mL IL-3 (Miltenyi Biotec), 20ng/mL G-CSF (Shenandoah, Warminster, Pennsylvania, USA), 10-4 M β-mercaptoethanol (Gibco), 50 µg/mL Gentamicin (Gibco) plus 10 µg/mL Ciprofloxacin (GenHunter, Nashville, Tennessee, USA). Stem regenin (SR) 1 and UM729 (StemCell Technologies) were added freshly to the media at a final concentration of 500nM.

HEK 293T cells were obtained from the American Type Culture Collection (ATCC, Manassas, Virginia, USA). Cells were cultured in DMEM (Gibco) supplemented with 10% heat-inactivated FBS (Sigma-Aldrich). Human AML cell lines were purchased from Leibniz Institute DSMZ-German Collection of Microorganisms and Cell Cultures (DSMZ, Braunschweig, Germany) and maintained as per the recommendations. Briefly, K562 cells were cultured in RPMI (Gibco) with 10% FBS (Sigma-Aldrich), OCI-AML2 and OCI-AML3 were maintained in alpha MEM (Gibco) supplemented with 20% FBS (Sigma-Aldrich).

Cells were cultured at 37 °C under an atmosphere of 5% CO2 at 0.3-1x10^6^/mL in 12 well plates or for larger-scale cell cultures in 6 well plates.

## **Colony Assays**

HSPCs were transduced with high titer lentivirus particles of shLuciferase or shLPIN1.1159, shLPIN1.452 and shLPIN1.957, respectively. After 5 days cells were sorted for bright fluorescence into methylcellulose (StemCell Technologies) supplemented with 50 µg/mL Gentamicin (Gibco). 0.5 mL per well were plated in 12 well plates using syringes with blunt end needles. After 10-14 days of culture in a humidity chamber, colonies were counted using an inverted microscope at 10-20x magnification.

## **Flow Cytometry and Cell Sorting**

Differentiation of HSPCs in liquid cultures was monitored using APC Mouse Anti-Human CD34 (BD Pharmingen), PE Mouse Anti-Human CD45RA (BD Pharmingen), PE/Cyanine5 Anti-Human CD11b (BioLegend, San Diego, California, USA) and APC/Cyanine7 Anti-Human CD14 (BioLegend). *In vivo* differentiation potential of HSPCs in transplanted NSGW41 was analyzed by staining bone marrow cells with APC Mouse Anti-Human CD45 (BD Pharmingen), PE-Cy™5 Mouse Anti-Human CD33 (BD Pharmingen), PE-Cy™7 Mouse Anti-Human CD19 (BD Pharmingen) and FITC Mouse Anti-Human CD3 (BD Pharmingen). AML cells in culture and from engrafted mouse bone marrows were stained for Pacific Blue™ anti-human CD45 Antibody (BioLegend), PE anti-human GPR56 Antibody (BD Pharmingen), APC Mouse Anti-Human CD34 (BD Pharmingen) or BV570 Mouse Anti-Human CD34 (BD Pharmingen), PE/Cyanine5 Anti-Human CD11b (BioLegend) and APC/Cyanine7 Anti-Human CD14 (BioLegend). Cells were sorted on a BD FACS Aria II (BD Pharmingen). Data acquisition was performed on a BD LSRII or BD Celesta flow cytometer (BD Pharmingen) equipped with a High throughput sampler (HTS) device and analyzed using BD FACS Diva 4.0 and BD FACS Diva 10, respectively. Further analysis was performed with BD FlowJo X software (Treestar Inc., Dartmouth, Arizona).

## **Proliferation Assay**

Primary cells and cell lines were transduced with shLuciferase or shLPIN1.1159, shLPIN1.452 or shLPIN1.957 using lentivirus. Cells were seeded at a density of 5-10x10^3^ per well in a 96 well plate. Cell counts and optionally CD34 surface expression were assessed at indicated time points *via* flow cytometric measurement of Ametrine fluorescent positive and APC positive cells with an HTS.

OCI-AML3 single clones created using the CRISPR/Cas9 system were seeded in a 96 well plate. Cell counts were assessed at indicated time points using flow cytometric measurement with an HTS and analyzed as described above.

## **Analysis of apoptosis**

10x binding buffer was added to the cells to be analyzed. Cells were stained for 15 minutes at room temperature with Annexin V-FITC (R&D Systems, Minneapolis, Nebraska, USA) and propidium iodide (PI) (Sigma-Aldrich). The proportion of viable, apoptotic and necrotic cells was assessed using flow cytometric measurement with an HTS and analyzed as described above.

## **Cell cycle analysis**

Cells were washed with PBS, resuspended and fixed with 10% formaldehyde (Boster, Pleasanton, California, USA) for 15 minutes at room temperature. For permeabilization cells were incubated for 10-15 minutes in a 37 °C water bath with 0.1% triton x-100 in 1% BSA (Sigma-Aldrich), followed by 5 minutes on ice with 0.05 % triton x-100 in 1% BSA (Sigma-Aldrich, St. Louis, Missouri, USA). Permeabilization buffer was removed, cells were carefully resuspended in 70% methanol while vortexing and incubated at -20 °C for 20 minutes. Cells were washed with cold PBS and stained with pyronin and DAPI (Sigma-Aldrich) or PI (Sigma-Aldrich) for 20 minutes. After washing with 1% BSA (Sigma-Aldrich) in PBS data were acquired on a BD LSRII or BD Celesta flow cytometer (BD Pharmingen) and analyzed as described above.

## Drug profiling using inhibitors of glycerophospholipid metabolism

For assessing the efficiency of small molecule inhibitors of glycerophospholipid metabolism in AML and hematopoiesis, primary AML samples (AML-661, AML-663, AML-346, AML-372 and AML 602) and HSPCSs were transduced with shRNA against *LPIN1* or *Luciferase*, and seeded at a density of 1x10^4^ cells per well in a 384 well format. Cells were incubated with small molecule inhibitors targeting CHKɑ including RSM-932A (MedChemExpress, Monmouth Junction, New Jersey, USA), ICL-CCIC-0019 (TargetMol, Wellesley Hills, Massachusetts, USA), EB-3P and EB-3D (generated and provided by Luisa C. López-Cara and Pilar M. Luque-Navarro) [^14^](https://sciwheel.com/work/citation?ids=17303672&pre=&suf=&sa=0), blocking CEPT1 using geranylgeraniol (Sigma), or nSMAse using GW4869 (Selleckchem) at increasing dosages for 5-7 days. At the day of readout, viable cells were counted by HTS-FACS. AML-661 and AML-602 were also stained with anti-CD34 antibody (BD#746987). Dose response profiles and CD34 surface expression were acquired using an LSR II flow cytometer equipped with an HTS. The intermediate concentration at which 50% of the cells remained viable (IC50) was calculated by fitting viable cell counts and concentrations to a sigmoidal model.

## Lentivirus production and transduction

Small hairpin (sh) oligo RNAs were selected based on optimized algorithms [^15^](https://sciwheel.com/work/citation?ids=44645&pre=&suf=&sa=0) and subcloned into a lentivector, in which a fluorescent marker (Ametrine) and the shRNA are driven by an MNDU3 promoter as described [^16^](https://sciwheel.com/work/citation?ids=6951527&pre=&suf=&sa=0). Oligo sequences used for knock down of *LPIN1* can be retrieved from Supp. Table 14. As control served a 96mer against Renilla luciferase namely shRen.713 (in the following called shLuc) which was a gift from Johannes Zuber [^15^](https://sciwheel.com/work/citation?ids=44645&pre=&suf=&sa=0) (Supp. Table 15). Oligos were amplified using EcoRI (Thermo Fisher Scientific) forward primer and XhoI (Thermo Fisher Scientific) reverse infusion primers (Supp. Table 13), followed by insertion of the sequence into an EcoRI (Thermo Fisher Scientific) and XhoI (Thermo Fisher Scientific) digested linearized vector using In-Fusion® Snap Assembly Master Mix (Takara, Kusatsu, Japan) according to the company’s recommendation.

*LPIN1A* full length (FL) cDNA sequence (NCBI NM_145693.4) ORF clone and an enzymatically impaired version of LPIN1 (ΔPAP) [^17^](https://sciwheel.com/work/citation?ids=325375&pre=&suf=&sa=0) were purchased from GenScript (GenScript, Piscataway, New Jersey, USA) and custom cloned into modified pmCherry-C1 Vector (Takara), in which the protein of interest is fused to the C-terminus of mCherry fluorescent protein, using 5’ BglII (Thermo Fisher Scientific) and 3’ EcoRI (Thermo Fisher Scientific) flanking sites. *LPIN1* FL and ΔPAP were also sub-cloned into the pLVX-EF1a-IRES-mCherry backbone, using SpeI (Thermo Fisher Scientific) and NotI (Thermo Fisher Scientific) restriction digestion and T4 ligation (Thermo Fisher Scientific).

High titer lentiviral particles were produced according to previously described protocols [^18^](https://sciwheel.com/work/citation?ids=6005680&pre=&suf=&sa=0). The following modifications were made: For generation of virus particles HEK293T cells were transfected using TurboFect transfection reagent (Thermo Fisher Scientific) for temporary packaging the cells with 7.5 μg lentiviral vector, 4.5 µg VSV-G plasmid and 15 μg psPAX2 packaging plasmid which was a gift from Didier Trono. Cells were cultured in DMEM (Gibco) supplemented with 3% FBS (Sigma-Aldrich). After 48 and 72 hours, supernatants containing virus particles were harvested. For maximum yield, particles were ultracentrifuged through a 20% sucrose (Sigma-Aldrich) cushion for 2 hours at 4°C and 29 000 rpm using a Sorvall WX Ultra 100 ultracentrifuge (Thermo Fisher Scientific). Virus particles were reconstituted in Opti-MEM (Gibco) and stored at -80°C until use. Virus titer was determined on OCI-AML3 cells in a 96 well plate on BD Celesta flow cytometer (BD Pharmingen) equipped with a HTS. For lentiviral transduction, primary cells were pre-incubated with 10µg/mL protamine sulfate (Sigma-Aldrich) for 30 min, followed by addition of lentiviral particles at a multiplicity of infection (MOI) of 20 directly to the culture. Cell lines were transduced by addition of polybrene (Sigma-Aldrich) and subsequent addition of lentiviral particles at MOI of 5-10. Cells and lentiviral particles were incubated for 12-16 hours under culture conditions before three times washing with PBS. 48-72 hours post infection gene transfer was assessed by flow cytometry.

## **CRISPR/Cas9 and generation of stable *LPIN1* or *GFP* knockout single clones**

Electroporation of cells was performed using the Neon transfection system (Invitrogen, Waltham, Massachusetts, USA). 1 µg single guide RNA (sgRNA) against *LPIN1* or green fluorescent protein (GFP) (Benchling, San Francisco, California, USA) were incubated with 1µg Cas9 protein (PNA Bio, Thousand Oaks, California, USA). 150x10^3^ cells per 10µL reaction were electroporated as described [^16^](https://sciwheel.com/work/citation?ids=6951527&pre=&suf=&sa=0) at 1700 mV, 10 ms and 3 pulses. Knockout efficiency was assessed by isolation of genomic DNA 4 days after electroporation using Qiagen DNeasy blood and tissue kit (Qiagen). A 500 bp target region around the cleavage site was amplified using the following primer pair BenchSgLpin1_2amp_fwd and BenchSgLpin1_2amp_rev (Supp. Table 14). Amplified sequences were gel purified and conferred to Sanger sequencing using either forward or reverse primers. Knockout efficiency was estimated using the Interference for CRISPR edits (ICE) tool from Synthego (https://ice.synthego.com, Synthego, Menlo Park, California, USA), which uses non-negative least squares regression to detect differences between input sanger sequences.

To generate single clones with stable *LPIN1* or GFP knockout (KO), OCI-AML3 were electroporated as described. On the second day after electroporation, they were seeded as single cells into 96 well plates using cell sorting. After a period of expansion, KO efficiency for each clone was assessed by target region amplification, sanger sequencing, and ICE analysis. Protein loss was validated *via* western blot.

## **Western Blot**

Total lysates were obtained by washing sorted cells with PBS, pelleting and subsequent lysis on ice for 30 minutes in a RIPA lysis buffer (Thermo Fisher Scientific) supplied with protease inhibitor cocktail (Sigma-Aldrich, St. Louis, Missouri, USA) and 2.5 U/mL Benzonase (Millipore, Burlington, Massachusetts, USA). After centrifugation at 13 000 rpm for 15 minutes at 4°C, supernatants from lysates were transferred into pre-chilled tubes. The protein concentration was measured using Bradford reagent and BSA standards (BioRad, Hercules, California, USA). Protein concentration between samples was adjusted with RIPA lysis buffer (Thermo Fisher Scientific), and NuPAGE™ LDS Sample Buffer (4x) (Invitrogen) was added. Protein was reduced at 90°C for 10 minutes and run on a precast gel. Protein was transferred onto a nitrocellulose membrane, blocked with 5% milk and incubated overnight at 4 °C with primary antibodies. Secondary antibodies, either HRP tagged anti-mouse or anti-rabbit secondary antibodies (1:5 000) (Jackson ImmunoResearch, West Grove, Pennsylvania, USA) were incubated with the membrane for 2 hours at room temperature. For membrane development, Amersham ECL Prime Western Blotting Detection Reagent kit (Amersham, Little Chalfont, England, United Kingdom) or Femto kit (Thermo Fisher Scientific) were used, followed by detection on GE Healthcare Life Sciences, Amersham Imager 600 (Amersham). The band intensities were quantified with ImageJ version 1.54g (U.S. National Institutes of Health, Bethesda, MD, USA).

Antibodies for western blotting were used at the following concentrations: anti-LPIN1 antibody (1:1 000) (Abcam, Cambridge, United Kingdom), anti-LPIN2 antibody (1:1 000) (Abcam), anti-LPIN3 antibody (1:500) (LSBio, Seattle, Washington, USA), anti-actin antibody (1:500) (Santa Cruz, Dallas, Texas, USA), anti-GAPDH antibody (1:2.500) (Biozol, Eching, Germany), and anti-Vinculin antibody (1:500) (Santa Cruz).

## **Immunofluorescence Imaging**

15x10^4^ HEK293T cells in 6 well plates were transiently transfected with the *LPIN1* overexpression vector or empty vector using 2 µg vector of interest and 4 µl TurboFect Transfection reagent (Thermo Fisher Scientific) in 175 µl Opti-MEM (Gibco). After 48 hours cells were trypsinized and 8x10^4^ cells per well were transferred in a 12 well plate containing autoclaved Poly-L-Lysine (Sigma-Aldrich) coated coverslips. Following overnight culture cells were washed with PBS and fixed in 4% paraformaldehyde (Boster) at 4 °C for 15 minutes. Coverslips were mounted on microscopy slides. Images were acquired with Volocity software (Perkin Elmer, Waltham, Massachusetts, USA) at 100x magnification with oil as immersion medium on a ERS-6 confocal microscope of the spinning disk type (Andor Technology, Belfast, Northern Ireland, United Kingdom) at The Nikon Imaging Center Heidelberg. Images were processed using Fiji and Lightroom Classic (Adobe, San Jose, California, USA).

## **Quantitative real-time polymerase chain reaction (q-RT-PCR)**

Cells designated for analysis by q-RT-PCR were either sorted for AM+ indicating successful transduction with shRNAs, or used directly, if gene transfer assessed by flow cytometry exceeded 80%. For extraction of high-quality RNA, a combination of Trizol reagent (Invitrogen) -chloroform (Sigma-Aldrich) purification according to the manufacturer’s recommendation followed by silica-membrane purification with the RNeasy Mini Kit (Qiagen) was used. RNA was reversely transcribed into cDNA using M-MLV-RT (Thermo Fisher Scientific). Q-RT-PCR was performed on a BioRad CFX96 Touch Real Time PCR detection system (BioRad) using SYBR mix reagent (BioRad). GAPDH served as endogenous control. Used primer pairs can be retrieved from Supp. Table 14.

## **Statistical analyses**

Statistical significance of lipidomics was tested using ordinary two-way ANOVA corrected for multiple comparison with Dunnett’s multiple comparison test with individual variances computed for each comparison. Data are shown as mean and SD.

For mRNA expression between AML subgroups, statistical significance was tested using ordinary one-way ANOVA corrected for multiple comparison with Tukey’s multiple comparison test with a single pooled variance. Survival probability was compared using a log-rank test and plotted using Kaplan-Meier curves. FACS data for proliferation, cell cycle, and apoptosis, and data from colony forming assays are shown as mean and SD, unpaired t-tests were used for comparison of two groups. For comparisons of more than one group with the control, ordinary one-way ANOVA and two-way ANOVA, respectively, corrected for multiple comparison with Dunnett’s multiple comparison test with a single pooled variance were applied. Results from xenotransplantation assays are represented by dot plots where horizontal bars indicate medians and statistical significance was tested using Mann-Whitney U test. If other statistical tests were used this is indicated in the figure legend.

**References**

[**1.**  ICGC/TCGA Pan-Cancer Analysis of Whole Genomes Consortium. Pan-cancer analysis of whole genomes. *Nature*. 2020;578(7793):82-93. doi:10.1038/s41586-020-1969-6](https://sciwheel.com/work/bibliography/8183995)

[2. Mölder F, Jablonski KP, Letcher B, et al. Sustainable data analysis with Snakemake. *F1000Res*. 2021;10:33. doi:10.12688/f1000research.29032.2](https://sciwheel.com/work/bibliography/10318268)

[3. Andrews S. FastQC: a quality control tool for high throughput sequence data. 2010.](https://sciwheel.com/work/bibliography/15884640)

[4. Martin M. Cutadapt removes adapter sequences from high-throughput sequencing reads. *EMBnet j*. 2011;17(1):10. doi:10.14806/ej.17.1.200](https://sciwheel.com/work/bibliography/827836)

[5. Dobin A, Davis CA, Schlesinger F, et al. STAR: ultrafast universal RNA-seq aligner. *Bioinformatics*. 2013;29(1):15-21. doi:10.1093/bioinformatics/bts635](https://sciwheel.com/work/bibliography/49324)

[6. Harrow J, Frankish A, Gonzalez JM, et al. GENCODE: the reference human genome annotation for The ENCODE Project. *Genome Res*. 2012;22(9):1760-1774. doi:10.1101/gr.135350.111](https://sciwheel.com/work/bibliography/43579)

[7. Ewels P, Magnusson M, Lundin S, Käller M. MultiQC: summarize analysis results for multiple tools and samples in a single report. *Bioinformatics*. 2016;32(19):3047-3048. doi:10.1093/bioinformatics/btw354](https://sciwheel.com/work/bibliography/2919136)

[8. Liao Y, Smyth GK, Shi W. featureCounts: an efficient general purpose program for assigning sequence reads to genomic features. *Bioinformatics*. 2014;30(7):923-930. doi:10.1093/bioinformatics/btt656](https://sciwheel.com/work/bibliography/148598)

[9. Love MI, Huber W, Anders S. Moderated estimation of fold change and dispersion for RNA-seq data with DESeq2. *Genome Biol*. 2014;15(12):550. doi:10.1186/s13059-014-0550-8](https://sciwheel.com/work/bibliography/129353)

[10. Huang DW, Sherman BT, Lempicki RA. Bioinformatics enrichment tools: paths toward the comprehensive functional analysis of large gene lists. *Nucleic Acids Res*. 2009;37(1):1-13. doi:10.1093/nar/gkn923](https://sciwheel.com/work/bibliography/43637)

[11. Ge SX, Jung D, Yao R. ShinyGO: a graphical gene-set enrichment tool for animals and plants. *Bioinformatics*. 2020;36(8):2628-2629. doi:10.1093/bioinformatics/btz931](https://sciwheel.com/work/bibliography/8106072)

[12. Özbalci C, Sachsenheimer T, Brügger B. Quantitative analysis of cellular lipids by nano-electrospray ionization mass spectrometry. *Methods Mol Biol*. 2013;1033:3-20. doi:10.1007/978-1-62703-487-6_1](https://sciwheel.com/work/bibliography/9860151)

[13. Paltauf F, Hermetter A. Strategies for the synthesis of glycerophospholipids. *Prog Lipid Res*. 1994;33(3):239-328. doi:10.1016/0163-7827(94)90028-0](https://sciwheel.com/work/bibliography/13678198)

[14. Schiaffino-Ortega S, Baglioni E, Mariotto E, et al. Design, synthesis, crystallization and biological evaluation of new symmetrical biscationic compounds as selective inhibitors of human Choline Kinase α1 (ChoKα1). *Sci Rep*. 2016;6:23793. doi:10.1038/srep23793](https://sciwheel.com/work/bibliography/17303672)

[15. Fellmann C, Hoffmann T, Sridhar V, et al. An optimized microRNA backbone for effective single-copy RNAi. *Cell Rep*. 2013;5(6):1704-1713. doi:10.1016/j.celrep.2013.11.020](https://sciwheel.com/work/bibliography/44645)

[16. Garg S, Reyes-Palomares A, He L, et al. Hepatic leukemia factor is a novel leukemic stem cell regulator in DNMT3A, NPM1, and FLT3-ITD triple-mutated AML. *Blood*. 2019;134(3):263-276. doi:10.1182/blood.2018862383](https://sciwheel.com/work/bibliography/6951527)

[17. Peterson TR, Sengupta SS, Harris TE, et al. mTOR complex 1 regulates lipin 1 localization to control the SREBP pathway. *Cell*. 2011;146(3):408-420. doi:10.1016/j.cell.2011.06.034](https://sciwheel.com/work/bibliography/325375)

[18. Lehnertz B, Zhang YW, Boivin I, et al. H3K27M/I mutations promote context-dependent transformation in acute myeloid leukemia with RUNX1 alterations. *Blood*. 2017;130(20):2204-2214. doi:10.1182/blood-2017-03-774653](https://sciwheel.com/work/bibliography/6005680)

[19. Győrffy B. Discovery and ranking of the most robust prognostic biomarkers in serous ovarian cancer. *Geroscience*. 2023;45(3):1889-1898. doi:10.1007/s11357-023-00742-4](https://sciwheel.com/work/bibliography/15313312)

[20. Győrffy B. Integrated analysis of public datasets for the discovery and validation of survival-associated genes in solid tumors. *Innovation (Camb)*. 2024;5(3):100625. doi:10.1016/j.xinn.2024.100625](https://sciwheel.com/work/bibliography/16492525)

[21. Győrffy B. Transcriptome-level discovery of survival-associated biomarkers and therapy targets in non-small-cell lung cancer. *Br J Pharmacol*. 2024;181(3):362-374. doi:10.1111/bph.16257](https://sciwheel.com/work/bibliography/16100258)

# Supplemental Tables

Supplemental Table 1: Reagent list

Supplemental Table 2: Multivariate analyses of the TCGA cohort

Supplemental Table 3: Genetic characterization of AML samples

Supplemental Table 4: Gene transfer in PDX samples

Supplemental Table 5: RNA-Seq data

Supplemental Table 6: Lipidomics of OCI-AML3 after LPIN1 knockdown

Supplemental Table 7: Lipidomics of 12 primary AML samples

Supplemental Table 8: Lipid nomenclature

Supplemental Table 9: Lipidomics sample information

Supplemental Table 10: Lipidomics metadata and quality control

Supplemental Table 11: Sciex Qtrap6500 settings and scan procedure

Supplemental Table 12: Sciex QTRAP5500 settings and scan procedure

Supplemental Table 13: Thermo QExactiv settings and scan procedure

Supplemental Table 14: Oligos

Supplemental Table 15: Plasmids

# **Figure Legends Supplementary Figures**

## **Supplemental Figure 1. High LPIN1 expression is associated with Leukemia with adverse course of disease and can be detected in AML cell lines and CD34+ HSPCs**

(A) Dot plot of *LPIN1* mRNA expression in normal karyotype (NK) AML samples with or without (WT) mutation of *DNMT3A*, *FLT3-ITD* and *NPM1*. Data is retrieved from the Leucegene project. Symbols represent individual samples, bars show median *LPIN1* expression. Statistical significance was tested using ordinary one-way ANOVA corrected for multiple comparison with Tukey’s multiple comparison test with a single pooled variance. (B) Column dot plot of *LPIN2* (left) and *LPIN3* (right) expression in 22 paired primary AML samples from the Leucege project at initial diagnosis and relapse. Paired t-tests. (C) Adapted Kaplan-Meier plot showing event free survival in dependence of *LPIN1* mRNA expression from three independent datasets, data was accessed *via* Kaplan-Meier Plotter [^19–21^](https://sciwheel.com/work/citation?ids=15313312,16492525,16100258&pre=&pre=&pre=&suf=&suf=&suf=&sa=0,0,0). (D) Cartoon illustrating the dual functions of LPIN1. HAT: histone acetyltransferase, G-3-P: glycerol-3-phosphate, LPA: lyso-p~~o~~hosphatidic acid, PA: phosphatidic acid, DG: diacylglycerol, TG: triacylglycerol. (E) Schematic sketch of the binding sites of shRNAs against *LPIN1* (created with wormweb). (F) Western blot showing LPIN1 protein in K562 upon lentiviral transduction with shRNAs against *LPIN1* or luciferase (shLuc). GAPDH was used as loading control. (G) Cell proliferation of K562 transduced with shLPIN1.1159 (blue) or shLPIN1.452 versus shLuc (lilac). Shown is the log10-fold increase in absolute cell counts per well until day 19 normalized to the eighth day of the culture. For each condition 8 replicates were started. Cells were counted by HTS-FACS. (H) Cell cycle analysis for OCI-AML3~~2~~ transduced with shLPIN1.1159 (blue), shLPIN1.452 (turquoise) or shLPIN1.957 (green) versus shLuc (lilac). Shown is the fraction of cells in G1, S and G2 phase. Three replicates were used per condition. Data are shown as mean+SD. Symbols represent individual replicates. Asterisks show results from ordinary one-way ANOVA corrected for multiple comparison with Dunnett’s multiple comparison test with a single pooled variance.

## **Supplemental Figure 2.** *LPIN1* is required to maintain the proliferative capacity and immature phenotype of AML cells

(A) KD efficiency of two different shRNAs against *LPIN1* compared to shLuc in AML-491 measured by q-RT-PCR (shLuc lilac, shLPIN1.1159 blue, shLPIN1.452 turquoise). Three replicates were used per condition. For better comparability between individual experiments, KD efficiencies were normalized to shLuc. Data are shown as mean+SD. (B) Proliferation of primary AML-491 cells transduced with shLPIN1.452 (turquoise) versus shLuc (lilac). Shown is the fold-increase in absolute cell counts per well normalized to the fourth day of culture. For each condition 5 replicates were started. Cells were counted by HTS-FACS. (C) Left panel: Representative FACS plots showing ametrine (AM+) expression and SSC-A of primary AML-491 cells transduced with shLPIN1.452 (bottom) versus shLuc (top) 24 weeks after transplantation into immunocompromised mice. Values indicate percentages. Right panel: Dot plot showing the engraftment of AM+ cells in the bone marrow of immunocompromised mice at week 24 after injection. Cells were either transduced with shLPIN1.452 (turquoise) or shLuc (lilac). The shLPIN1 group consisted of 4 mice, the shLuc group of 6 mice represented by symbols. The gene transfer in injected cells on day 4 was 87.1% in the shLPIN1 group and 90.4% in the control. Mann-Whitney U test. (D) Left panel: Representative FACS plots showing CD34 expression and SSC-A of primary AML-491 cells transduced with shLPIN1.452 (bottom) versus shLuc (top) 24 weeks after transplantation into NSG mice. Values indicate percentages. Right panel: Dot plot showing the percentage of CD34+ cells of engrafted AM+ cells. Cells were either transduced with shLPIN1.452 (turquoise) or shLuc (lilac). The shLPIN1 group consisted of 4 mice, the shLuc group of 6 mice represented by symbols. Mann-Whitney U test. (E) Expression of *LPIN1* mRNA in FACS sorted AM+ cells harvested from the bone marrow of mice injected with AML-491 cells transduced with shLPIN1.1159 (blue) versus shLuc (lilac) at 21 weeks post transplantation. Each group consisted of 7 mice represented by symbols. (F) Representative FACS plots illustrating the gating scheme applied to analyze CD34 and CD11b expression of primary AML-491 cells transduced with shRNA against *LPIN1* or shLuc. Viable cells were selected by their forward and side scatter profile followed by doublet exclusion using FSC-H and FSC-W. AM+ cells were identified and their surface expression of CD34 and intensity of CD11b were assessed. (G) Left panel: Representative histograms showing CD11b intensity of primary AML-491 cells transduced with shLPIN1.452 (bottom) versus shLuc (top) 24 weeks after transplantation in NSG mice. Values indicate percentages. Left panel: left: dot plot showing the fraction of CD11b+ cells among the engrafted Ametrine+ cells 24 weeks after transplantation of mice with AML-491 cells transduced with shLPIN1.452 (green) versus shLuc (lilac) in %. The shLPIN1 group consisted of 4 mice, the shLuc group of 6 mice represented by symbols. Mann-Whitney U test. Middle: Dot plot showing the mean fluorescence intensity (MFI) of CD11b PerCP among Ametrine+ cells 24 weeks after transplantation of NSG mice with AML-491 transduced with shLPIN1.452 (blue) versus shLuc (lilac). Unpaired t-test. Right: Dot plot showing the frequency of CD11b and CD14 co-positive cells among engrafted Ametrine+ cells after transplantation of mice with AML-491 cells transduced with shLPIN1.452 (blue) versus shLuc (lilac). Mann-Whitney U test.

## **Supplemental Figure 3. CD34 bright HSPCs respond highly sensitive to *LPIN1* suppression**

(A) KD efficiency of three different shRNAs against *LPIN1* compared to shLuc in HSPCs measured by q-RT-PCR (shLuc lilac, shLPIN1.1159 blue, shLPIN1.452 turquoise, shLPIN1.957 green). Three replicates were used per condition. For better comparability between individual experiments, KD efficiencies were normalized to shLuc. Data are shown as mean+SD. (B) Proliferation curves for HSPCs transduced with shLPIN1.1159 (blue) or shLPIN1.452 (turquoise) versus shLuciferase (lilac). Shown is the fold-increase in absolute cell counts per well on day 11 and day 15 normalized to the fifth day of the culture. For each condition 6 replicates were started. Cells were counted by HTS-FACS. (C) Colony forming cell assay of pooled HSPCs, which were transduced with shLPIN1.1159 (blue) or shLPIN1.452 (turquoise) versus shLuc (lilac) as negative control. Ametrine positive cells were directly sorted into methylcellulose 72 hours post transduction. 200 cells were plated per well and colonies were counted 10 days post plating. BFU-E: burst-forming unit-erythrocyte, GEMM: granulocyte, erythrocyte, megakaryocyte, macrophage, G: granulocyte, M: macrophage, GM: granulocyte/macrophage. (D) Top: Sorting strategy for AM+ HSPCs on CD34 and CD45RA expression; bottom: Proliferation of 4-way sorted HSPCs transduced with shLPIN1.1159 (blue) or shLPIN1.452 (turquoise) versus shLuc (lilac). Shown is the fold-increase in absolute cell counts per well as indicated. For each condition 10 replicate wells were seeded. Cells were counted by HTS-FACS.

## **Supplemental Figure 4.** RNA-seq and single CRISPR/Cas9 LPIN1 knockout clones highlight the enzymatic function of LPIN1

(A) Cartoon illustrating experimental setup of the RNA-Seq experiment. See Materials and methods for details. (B) Bar plot showing *LPIN1* expression in the RNA-Seq dataset in OCI-AML3 (left) and HSPCs (right) upon KD with shRNAs against *LPIN1* or *Luciferase*. Symbols represent replicates. Ordinary one-way ANOVA corrected for multiple comparison with Dunnett’s multiple comparison test with a single pooled variance. (C) Volcano plot showing the log2-fold changes (x-axis) and transformed p values (y-axis) of genes in HSPCs 48 hours after lentiviral transduction with shLPIN1.1159 or shLPIN1.957 versus shLuc. Data points plotted in gray represent genes without significant regulation. Data points highlighted by blue points represent significantly downregulated genes (p ≤ 0.05, log2-fold change (log2FC) shLPIN1/shLuc > 0), while data points highlighted by red dots represent significantly upregulated genes (p ≤ 0.05, log2FC shLPIN1/shLuc > 0). Not all gene symbols are displayed due to space limitations. (D) Western blot showing *LPIN1* expression of OCI-AML3 single clones. Actin was used as loading control. Numbers below the Western Blot indicate remaining LPIN1 protein and thereby knock out (KO) efficiency for *LPIN1* after KO using CRISPR/Cas9 normalized to a sgGFP single clone as control. (E) Bar plot showing proliferation of different OCI-AML3 sgLPIN1 single clones after KO using CRISPR/Cas9 compared to sgGFP single clones (lilac) as control. Shown is the fold-increase in absolute cell counts per well on day 11 normalized to the day when cells were taken in culture. For each sgLPIN1 clone 6 replicates were started. For sgGFP 8 single clones at 6 replicates were started as a reference. Cells were counted by HTS-FACS. Data is given as mean+SD. (F) Illustration of the LPIN1 protein domains. N-Lip: N-terminal lipin, NLS: nuclear localization signal, TAD: transcription factor activator domain, DXDXT: motif required for enzymatic PAP activity, LXXIL: transcriptional coactivator motif required for interaction with transcription factors like PPARG, C-Lip: C-terminal lipin. (G) Immunofluorescence images of HEK293T cells upon transfection with *LPIN1* full length (FL), *LPIN1* ΔPAP, or empty vector (ev), showing DAPI core staining and fluorescence of pmCherry-fused LPIN1.

## **Supplemental Figure 5. Lipid classes and lipid composition in OCI-AML3 and primary AML samples**

(A) Bar plot showing the distribution of lipid classes of OCI-AML3 after transduction with shLPIN1.452 (turquoise) or shLPIN1.957 (green) versus shLuc (lilac) measured by mass spectrometry. For each condition 4 replicates were measured. Data are shown as mean. Symbols represent individual replicates. PS: phosphatidylserine, PS O-: ether-linked phosphatidylserine, PI: phosphatidylinositol, PI O-: ether-linked phosphatidylinositol, PG: phosphatidylglycerol, PG O-: ether-linked phosphatidylglycerol, PA O-: ether-linked phosphatidic acid, Cer: creamide, SM: sphingomyelin, HexCer: hexosyl ceramide, Hex2Cer: di-hexosyl ceramide, Chol: cholesterol, CE: cholesterol ester, DG: diacylglycerol, TG: triacylglycerol. (B) Bar plot showing changes in phospholipid composition regarding the number of double bonds of PA in OCI-AML3 after transduction with shLPIN1.452 (turquoise) or shLPIN1.957 (green) versus shLuc (lilac) measured by mass spectrometry. For each condition 4 replicates were measured. Data are shown as mean. Symbols represent individual replicates. PA: phosphatidic acid. (C) Heatmap showing correlation of relative lipid class abundance in primary AML samples. Lipid abundance was measured by mass spectrometry. Colors indicate positive or negative Pearson correlation coefficients. (D) XY-plot displaying the relative abundance of phosphatidylcholine and phosphatidylcholine-ether, phosphatidylethanolamine and cholesterol, sphingomyelin and cholesterol, and phosphatidylcholine and ceramide, measured by mass spectrometry, in mol%. Pearson coefficient was calculated and a simple linear regression model using a 95% confidence interval was applied. (E) Principal component analysis (PCA) plot showing PC1 and PC2. Points represent primary human AML samples with indicated sample IDs. See Supplemental Table 3 for sample information. The shaded area is characterized by similar lipid composition and comprises all four samples with NPM1c/FLT3-ITD co-mutations.

## Supplemental Figure 6. LPIN1 and choline pathway inhibitors in AML.

(A) Dose-response curves of five PDX AML samples (AML-602, AML-661, AML-663, AML-346, AML-372) versus healthy hematopoietic stem and progenitor cells (HSPCs; CD34+ #1) treated with geranylgeraniol (GG) and GW4869. Cell numbers were normalized to DMSO vehicle alone. For calculation of half-maximal inhibitory concentrations (IC50), concentrations were transformed as log(µM+0.001). DMSO vehicle was set at 0 for GG and at -1 for GW. Curves were fitted using a sigmoidal model. The IC50s were not reached. (B) Dot plot of *LPIN1* mRNA expression from the Leucegene cohort (logRPKM). Symbols represent individual samples, and bars indicate median *LPIN1* expression. Statistical significance was tested using ordinary one-way ANOVA corrected for multiple comparisons with the Holm-Sidak test. Abbreviations: CD34: CD34+ HSPCs; NK_*FLT3ITD*_*NPM1c*: normal karyotype samples with mutations in *nucleophosmin 1c* (*NPM1c*) and internal tandem duplication (ITD) in *fms-related tyrosine kinase 3* (*FLT3*); complex_*TP53*_mol_mut: complex karyotype samples with mutations in *tumor protein 53* (*TP53*); complex_*TP53*_mol_wt: complex karyotype samples without *TP53* mutations; NK_*FLT3ITD*_*NPM1*wt: normal karyotype samples with *FLT3-ITD* without *NPM1c* mutations; *NPM1c*_*FLT*3wt: samples with *NPM1c* mutations without *FLT3-ITD* or other *FLT3* mutations; t(8;21): translocation of chromosome 8 to chromosome 21; *PML*::*RARA*: reciprocal *translocation of retinoic acid receptor ɑ* (*RARA*) with *promyelocyte leukemia gene* (*PML*). (C) XY plot showing expression of choline kinase ɑ (*CHKA*) versus *LPIN1* (normalized log RPKM) from the Leucegene cohort. Highlighted are HSPCs (CD34, lilac), normal karyotype samples with *FLT3-ITD* (NK F, blue), samples with t(8;21) (yellow), and samples with *PML::RARA* (green).
