# Supplementary figures and images for: Phosphatidic acid phosphatase LPIN1 in phospholipid metabolism and stemness in hematopoiesis and AML
Source: Hemasphere. 2025 Apr 22;9(4):e70118. doi: 10.1002/hem3.70118 (PMC12012646; doi:10.1002/hem3.70118)

Supplemental Figure 1

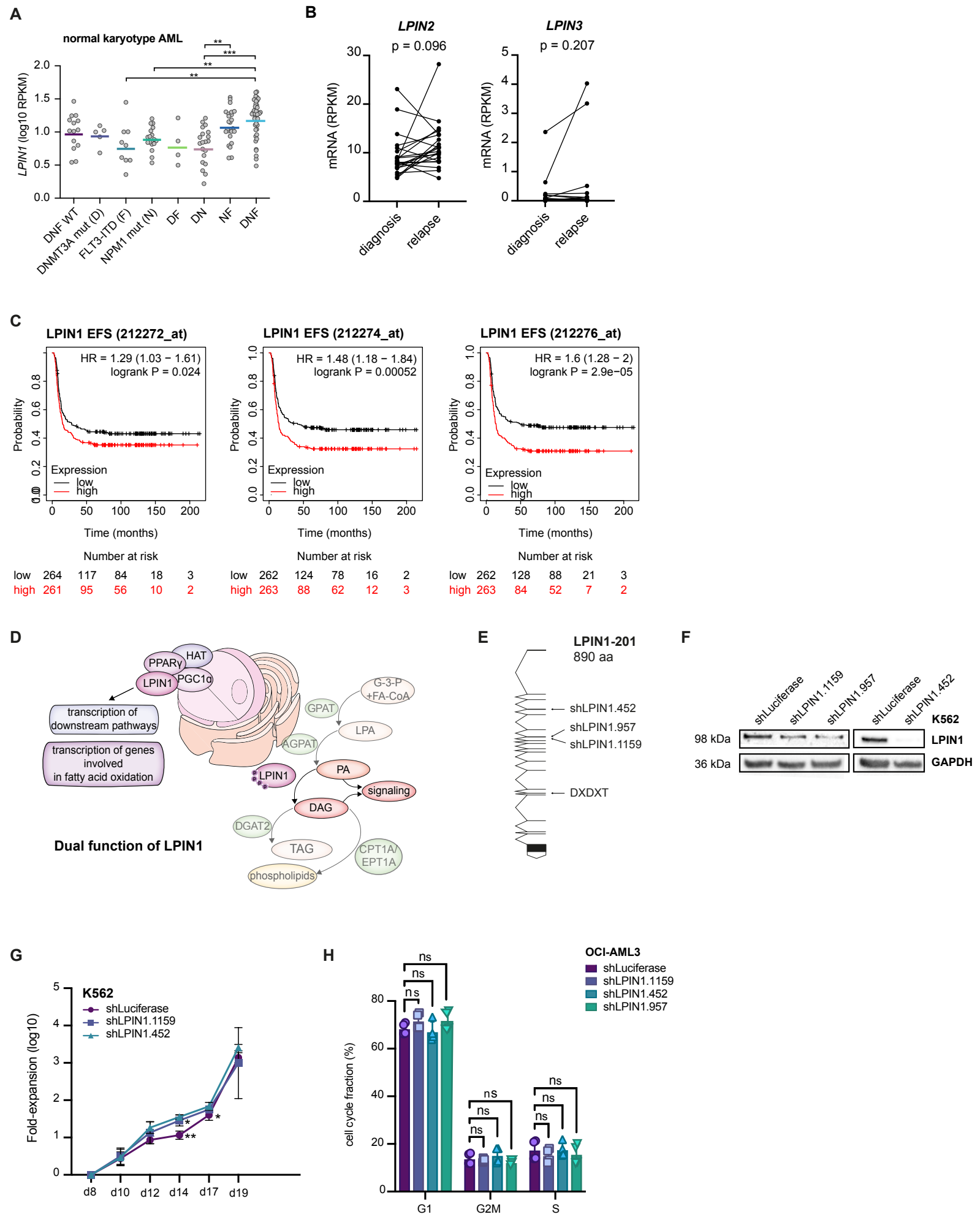

Supplement: Supplementary file 17 — Supporting information. [file HEM3-9-e70118-s012.pdf]

Supplemental Figure 2

A

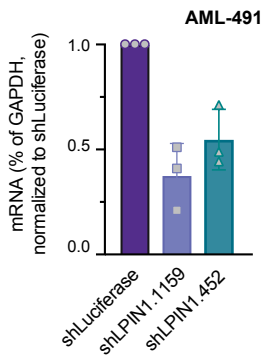

B

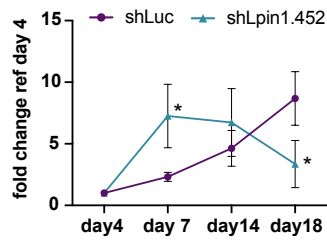

C

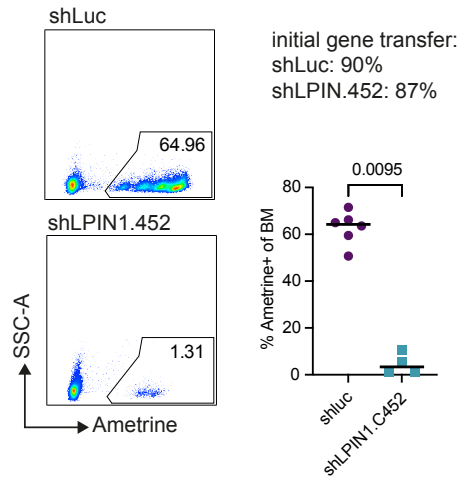

D

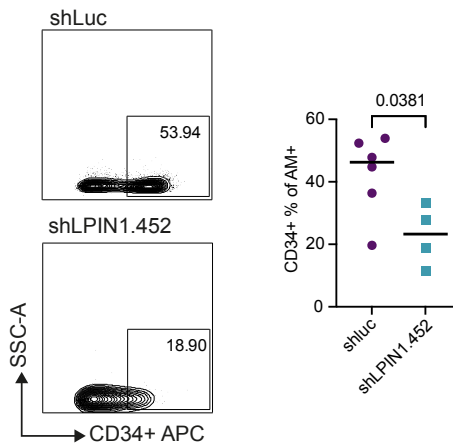

E

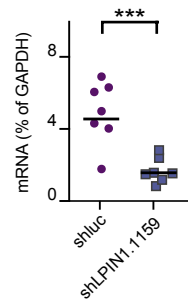

F

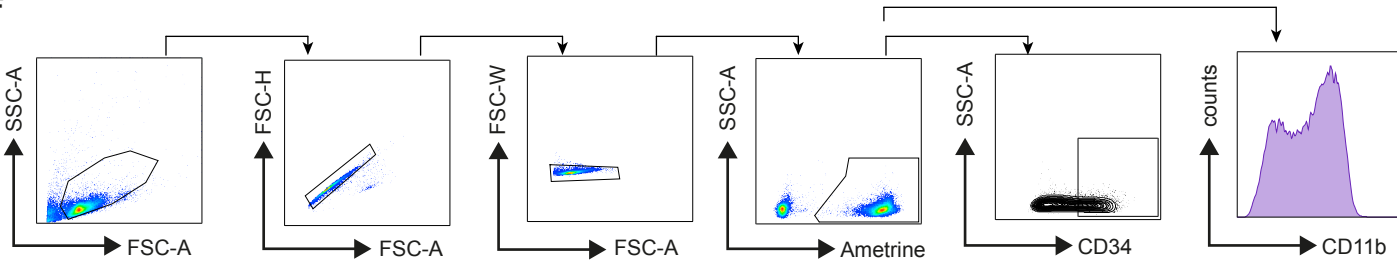

G

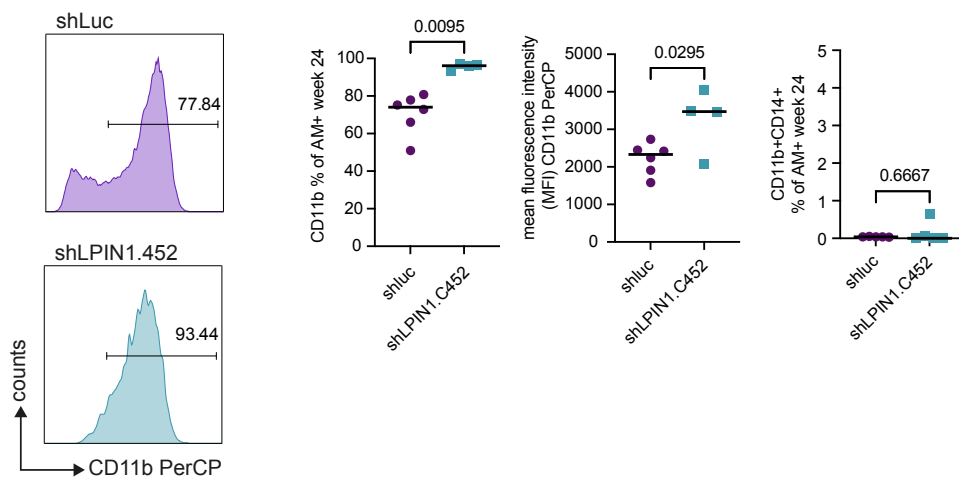

Supplement: Supplementary file 18 — Supporting information. [file HEM3-9-e70118-s018.pdf]

Supplemental Figure 3

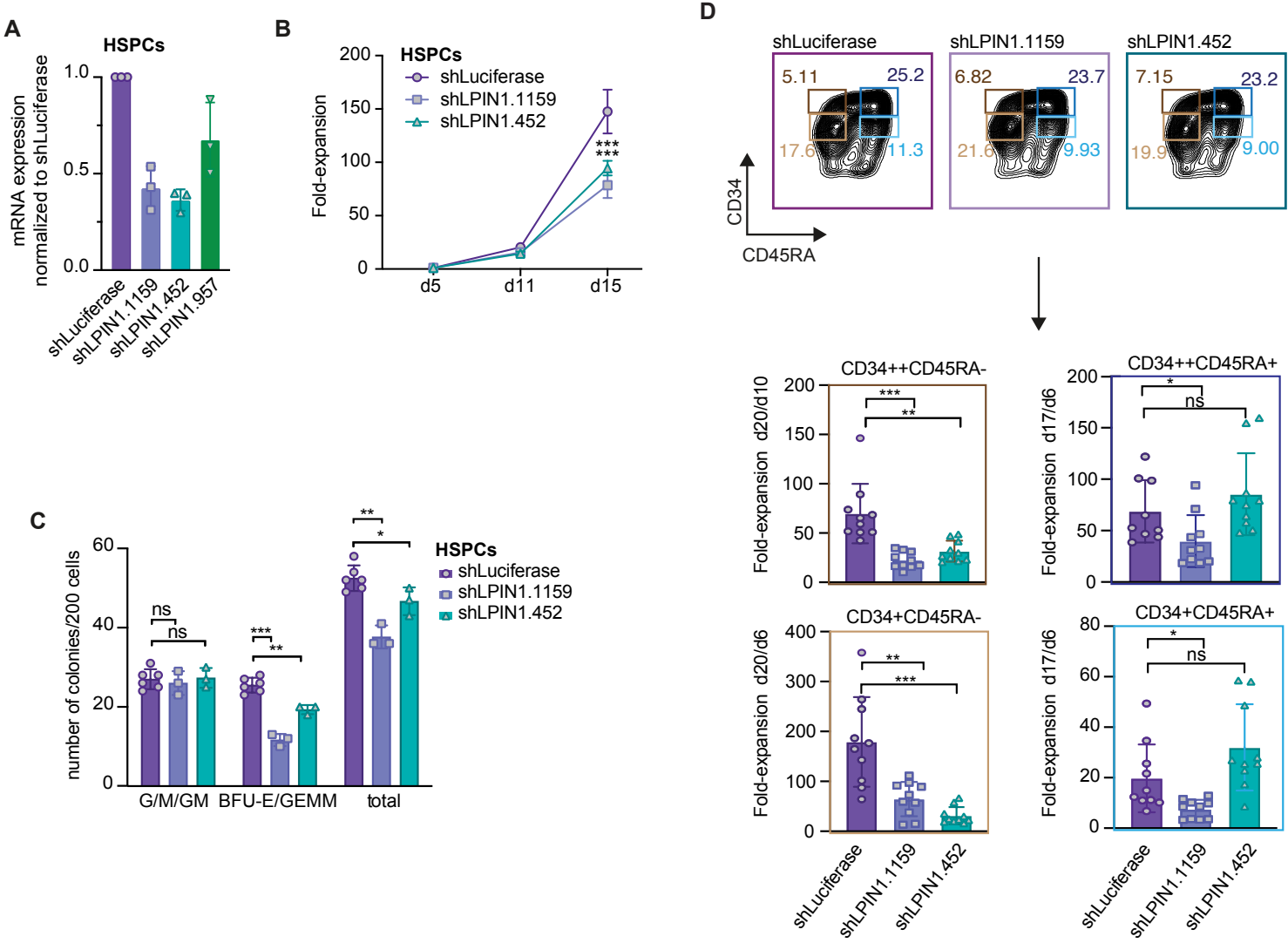

Supplement: Supplementary file 19 — Supporting information. [file HEM3-9-e70118-s016.pdf]

Supplemental Figure 4

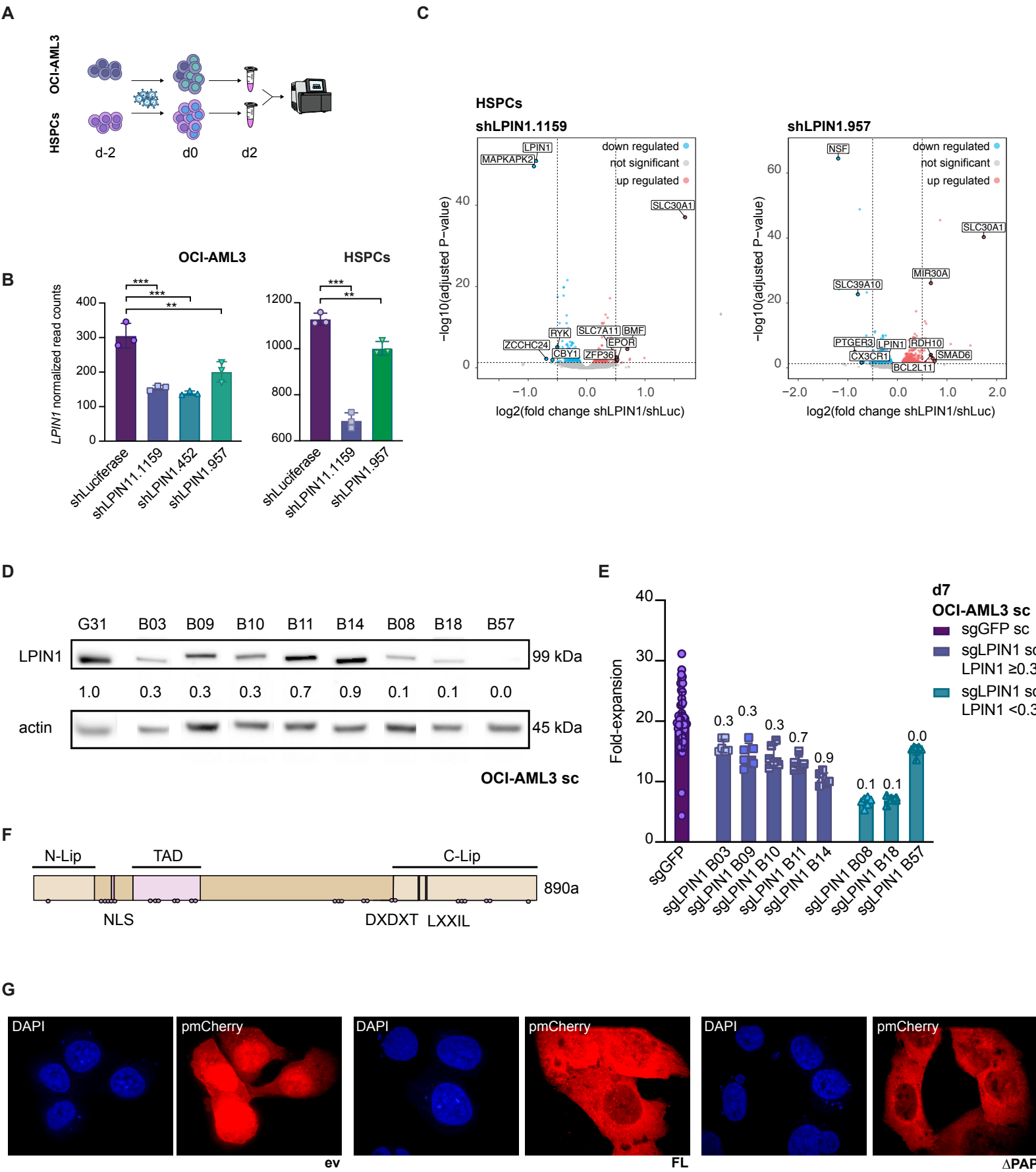

Supplement: Supplementary file 20 — Supporting information. [file HEM3-9-e70118-s014.pdf]

Supplemental Figure 5

A

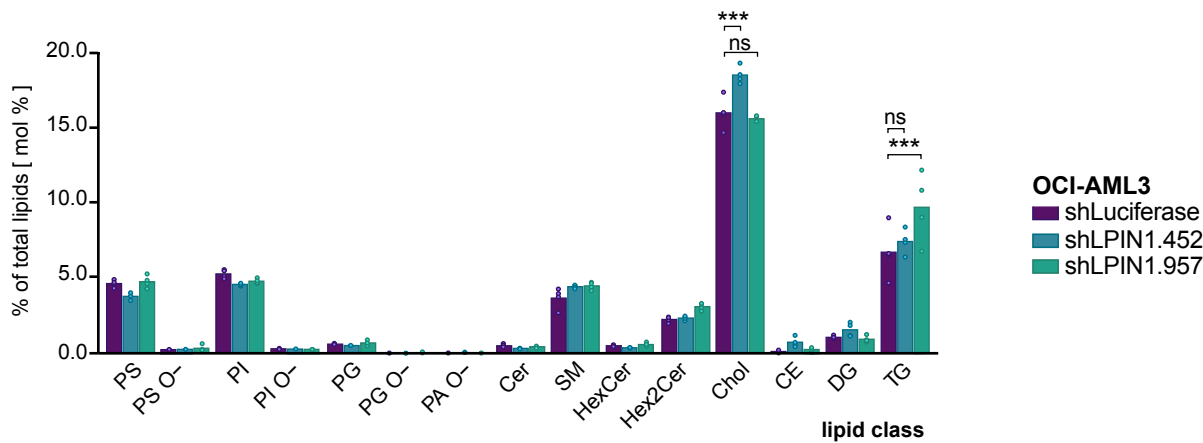

B

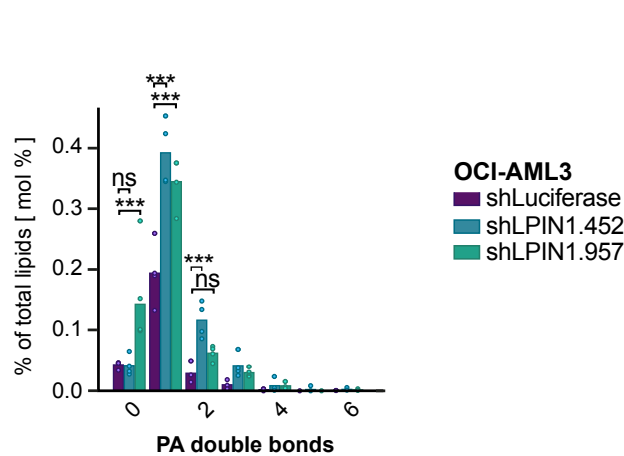

C

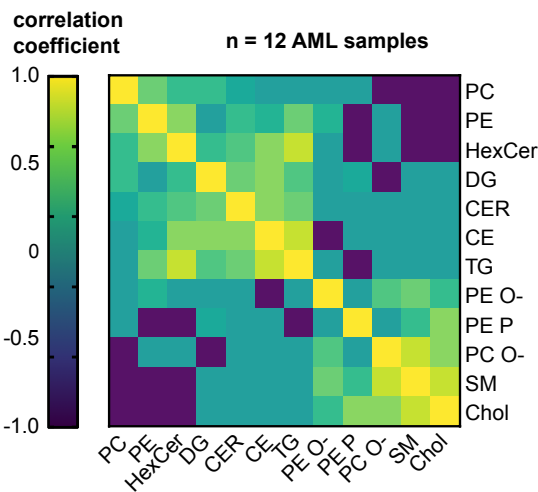

D

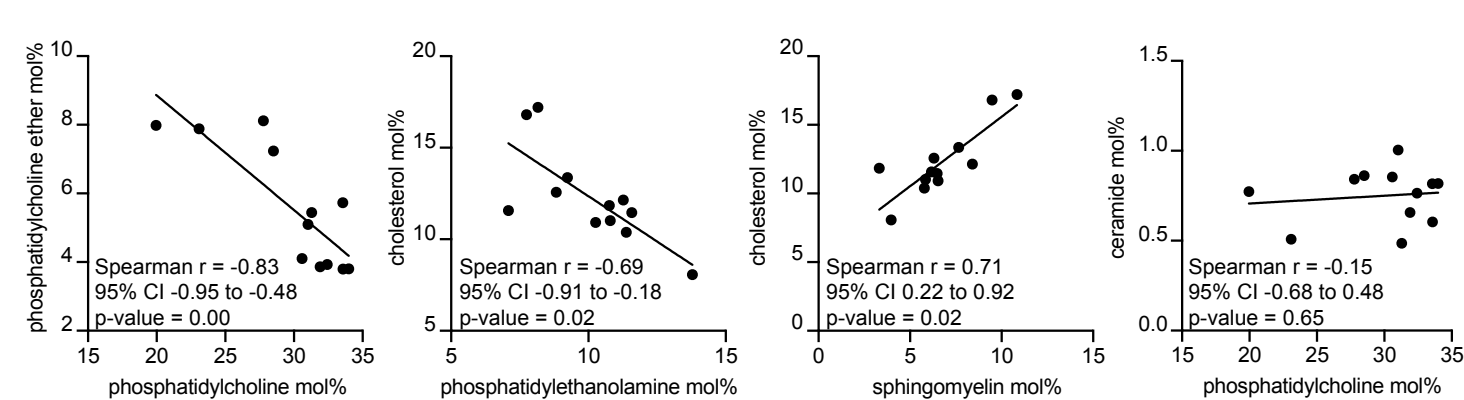

E

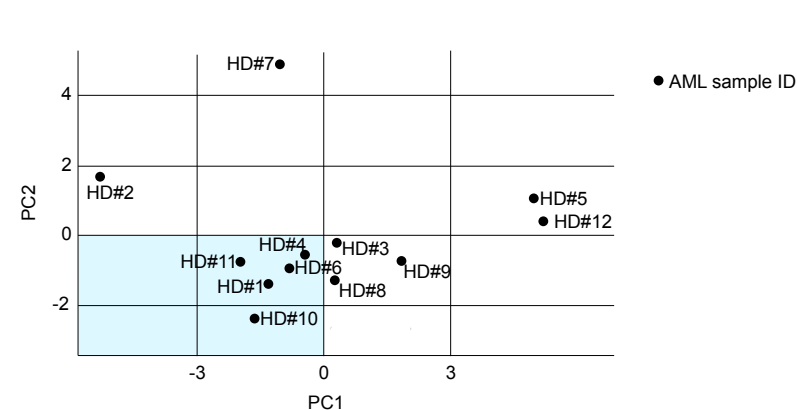

Supplement: Supplementary file 21 — Supporting information. [file HEM3-9-e70118-s004.pdf]

Supplemental Figure 6

A

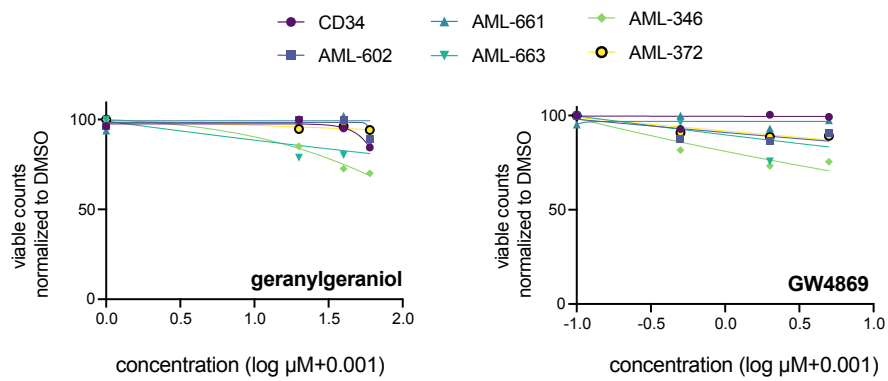

B

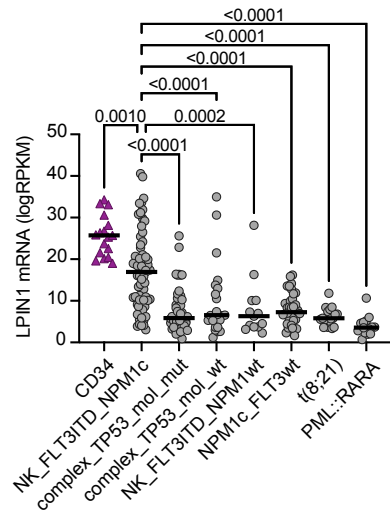

C

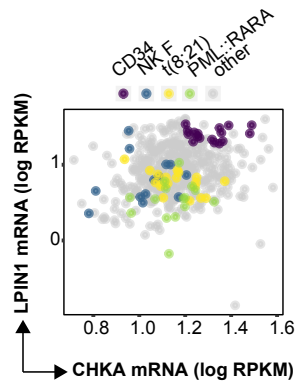

Supplement: Supplementary file 22 — Supporting information. [file HEM3-9-e70118-s001.pdf]
